# Pulmonary hypertension secondary to Abernethy malformation with left inferior vena cava: a case report and literature review

**DOI:** 10.3389/fmed.2025.1604853

**Published:** 2025-08-22

**Authors:** Xiaoxue Zhang, Mingyu Peng, Ruichen Ren, Wenting Li, Qingyuan Zhao, Chengcheng Qi, Yang Zhang

**Affiliations:** Department of Radiology, Qilu Hospital of Shandong University, Jinan, Shandong, China

**Keywords:** Abernethy malformation, congenital extrahepatic portosystemic shunt, pulmonary hypertension, anomalies of inferior vena cava, nodular liver lesions

## Abstract

**Background:**

Abernethy malformation is a rare condition in which the portomesenteric blood drains into systemic circulation, bypassing the liver. With advancements in imaging techniques and increased awareness of this malformation, there has been a growing number of reported cases in recent years. We present a case report and literature review in an effort to further the understanding of Abernethy malformation.

**Case presentation:**

We report a 21-year-old male presenting with pulmonary hypertension (PH) and right heart enlargement for 7 days. Portal CT angiography (CTA) revealed a vessel communication between the portal vein (PV) and the IVC, located on the left side of the abdominal aorta below the renal vein, and multiple nodular liver lesions (NLL). Finally, Abernethy malformation type II was diagnosed, which is extremely rare due to the absence of polysplenia while co-existing with anomalies of inferior vena cava (AIVC). He was discharged with stable vital signs on symptomatic therapy.

**Conclusion:**

Abernethy malformation presents with a range of clinical manifestations. It should be considered in patients with unexplained PH.

## Introduction

Abernethy malformation, or congenital extrahepatic portosystemic shunt (CEPS), is a rare condition in which the portomesenteric blood bypasses the liver and drains directly into systemic circulation. John Abernethy first reported it in 1793 (1). Morgan and Superina (2) classified Abernethy malformation into two types. Type I refers to the total aplasia of intrahepatic portal branches with complete extrahepatic shunting of portal blood into the systemic circulation. Type I is further divided into type Ia (the splenic vein and superior mesenteric vein drain separately into a systemic vein) and type Ib (the splenic vein and superior mesenteric vein drain together after joining to form a common trunk). Type II refers to hypoplastic intrahepatic portal branches with partial extrahepatic shunting of portal blood into the systemic circulation.

Abernethy malformation presents with diverse clinical manifestations and is associated with various congenital anomalies, including AIVC (3). Notably, polysplenia demonstrates a particularly high co-occurrence rate with AIVC in these cases (4). A previous report by Samir Shakya et al. (5) described a case of Abernethy malformation without polysplenia, accompanied by AIVC, in a pediatric patient. Our patient is an adult male with such a condition, along with PH and NLL.

## Case presentation

**FIGURE 1:**
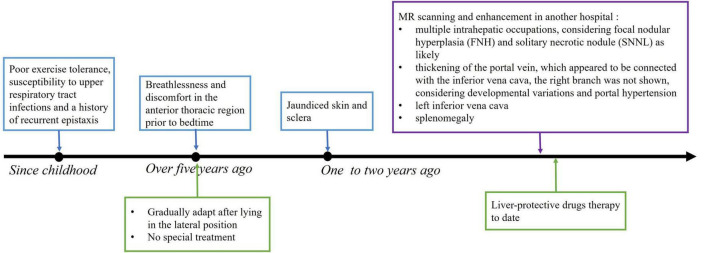
The timeline of the history.

A 21-year-old male presenting with PH and right heart enlargement for 7 days. His symptoms began over 5 years ago with breathlessness and anterior chest discomfort occurring primarily before bedtime. These symptoms gradually resolved after lying in a lateral position, and no specialized treatment was administered. PH and right heart enlargement were incidentally identified on an echocardiogram performed as part of a pre-employment medical examination 7 days ago. The physical examination showed that his sclera appeared slightly jaundiced. The timeline of the history is shown in Figure 1.

Laboratory tests showed decreases in white blood cell (3.23 × 10^9^/L; reference range, 3.5–9.5 × 10^9^/L), platelet counts (93 × 10^9^/L; reference range, 125–350 × 10^9^/L) and serum albumin levels (36.7 g/L; reference range, 40–55 g/L), and an increase in glutamate dehydrogenase (7.9 μmol/L; reference range, < 7.4 μmol/L), total bilirubin (35.1 μmol/L; reference range, 5.0–21.0 μmol/L), direct bilirubin (12.3 μmol/L; reference range, < 6.0 μmol/L), indirect bilirubin (22.8 μmol/L; reference range, 2.0–15.0 μmol/L), cholyglycine (42.34 μmol/L; reference range, < 2.7 μmol/L) and homocysteine (45.4 μmol/L; reference range, < 15.0 μmol/L). Activated partial thromboplastin time ratio was 1.33 (reference range 0.80–1.20) and fibrinogen was 1.81 (reference range 2.00–4.00). Urine protein was + – (reference –) and urobilinogen was 4 + (reference – or + –).

**FIGURE 2:**
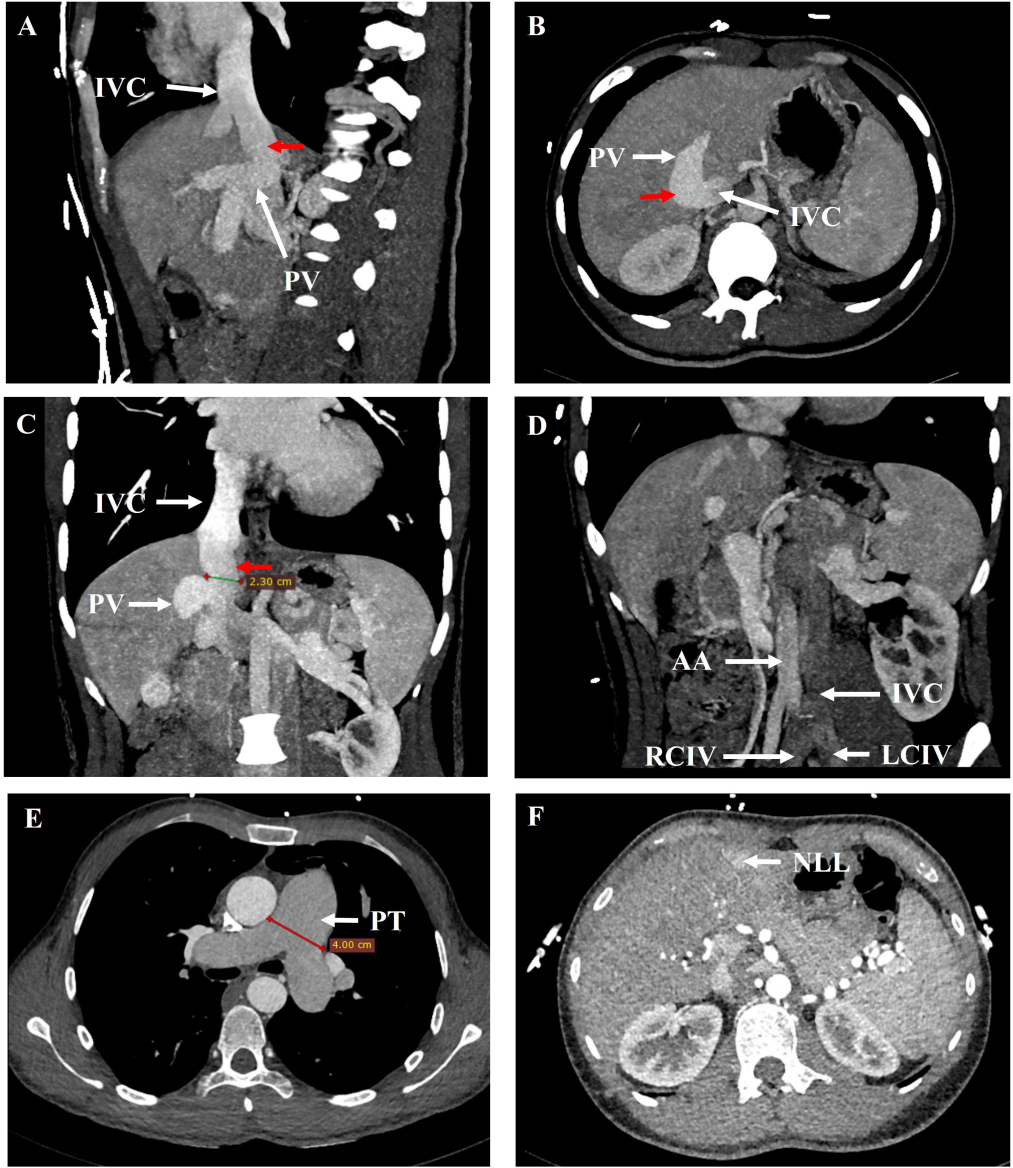
21 years-old male with Abernethy malformation type II, left ICV, and NLL. **(A)** Sagittal, **(B)** axial and **(C)** coronal maximum intensity projection image from contrast-enhanced CT revealed a vessel communication (red arrow) between the PV and the IVC. **(D)** Oblique coronal maximum intensity projection image demonstrated the IVC was on the left side of the abdominal aorta. **(E, F)** Arterial phase axial images from contrast-enhanced CT showed the dilated pulmonary trunk and the nodular liver lesions (NLL). AA, abdominal aorta; IVC, inferior vena cava; LCIV, left common iliac vein; NLL, nodular liver lesions; PV, portal vein; PT, pulmonary trunk; RCIV, right common iliac vein.

Echocardiography revealed the dilated right atrium (area: 23 cm^2^) and right ventricle (mid-cavity diameter: 34 mm, basal diameter: 44 mm), widening of the pulmonary trunk (31 mm), and severe PH. Pulmonary artery systolic pressure of about 82 mmHg estimated by the tricuspid regurgitation pressure gradient method (see Supplementary Data Sheet 1–S1). CT pulmonary angiogram and portal CTA demonstrated that the pulmonary trunk was dilated with a diameter of approximately 4.0 cm. The portal vein was dilated, with a diameter of approximately 1.6 cm. The left and right branches of the portal vein were poorly visualized. Additionally, splenomegaly was noted. An arcuate bridging vessel was observed communicating between the PV and the IVC, with a diameter of approximately 2.3 cm (Figure 2). The IVC was on the left side of the abdominal aorta below the renal vein (Figure 2). The liver displayed multiple enhanced nodules in the arterial phase (Figure 2). Rheumatologic serology, echocardiography, virological testing and CT pulmonary angiogram excluded connective tissue diseases, congenital heart disease, HIV infection, left heart disease, chronic lung disease, and chronic thromboembolic disease as potential etiologies of PH. Abernethy malformation type II with left IVC, PH and NLL was considered. A right heart catheterization confirmed precapillary PH with a mean pulmonary arterial pressure of 42 mmHg, pulmonary arterial wedge pressure of 3 mmHg, pulmonary vascular resistance of 4.88 Wood units, right atrial pressure of 4 mmHg, cardiac output of 8.0 L/min, and a cardiac index of 4.57 L/min/m^2^ (see Supplementary Data Sheet 1–S2).

The patient was administered ambrisentan and tadalafil targeted therapy for PH and was subsequently discharged with stable vital signs. Following discharge, oral medication was continued.

## Discussion

Abernethy malformation is a rare condition in which the portomesenteric blood drains into systemic circulation, bypassing the liver. Portal CTA showed that the left and right branches of the portal vein were poorly visualized and an arcuate bridging vessel was observed communicating between the PV and the IVC. According to the classification proposed by Morgan and Superina (2), these features are classified as Abernethy malformation type II. Of note, this case represents an exceptionally rare clinical occurrence of Abernethy malformation in an adult male with absence of polysplenia and coexistence of AIVC.

Abernethy malformation is a rare condition, with an increasing number of case reports in recent years due to advances in imaging techniques and greater awareness of this malformation. The literatures about Abernethy malformation published before September, 2024 were systematically collected. We searched PubMed, Web of Science, Embase and Cochrane databases using the search terms “Abernethy malformation*,” “congenital absence of portal vein,” “congenital absence of the portal vein,” “congenital extrahepatic portosystemic shunt*,” “congenital extrahepatic portocaval shunt*,” “congenital extrahepatic shunt*.” The following studies were excluded: those involving patients with clinical problems outside the target group (e.g., surgical shunts), fetal cases, autopsy cases, duplicate cases, literature such as conference abstracts and reviews, and studies on animals. A total of 295 studies (Supplementary Data Sheet 1–S3) involving 341 Abernethy malformation patients were included. The classification, gender, age, reported region, associated congenital diseases and complications, and mortality cases of the included patients were reported as case numbers. Intergroup differences were assessed by chi-squared (χ^2^) test and Fisher’s exact test, with *P* < 0.05 considered statistically significant. SPSS 27.0 software was used for statistical analyses.

There were 170 cases of type I, 169 cases of type II, and 2 cases of unknown type; 169 males, 169 females, and 3 patients with unknown gender or transgender; 221 children (≤ 18 years old) and 120 adults (> 18 years old) (Table 1); 181 cases in Asia, 150 cases in Europe and North America, and 10 cases in other regions. Aligning with the review by Kumar P et al. (6), there is no clear sex and age preponderance. Furthermore, among the Type I patients, there were 19 cases of Type Ia, 122 cases of Type Ib, and 29 cases of an unknown type.14 patients initially diagnosed as Type I were subsequently reclassified as Type II following further diagnostic procedures, including balloon occlusion angiography (7 cases), angiography (3 cases), liver biopsy (3 cases), and intraoperative findings (1 case).

**TABLE 1:** Comparison of gender and age distributions by Abernethy Malformation type.

Type	Gender			*P* value	Age		*P* value
	Male (*n* = 169)	Female (*n* = 169)	Unknow or transgender (*n* = 3)		Child (*n* = 221)	Adult (*n* = 120)	
I (*n* = 170)	77	90	3	0.190^a^	116	54	0.238^b^
II (*n* = 169)	90	79	0		105	64	
Unknow (*n* = 2)	2	0	0		0	2	

*^a^*Chi-square test comparing type (Type I, Type II) and gender (male, female);

*^b^*Chi-square test comparing type (Type I, Type II) and age (children, adult).

Abernethy malformation can cause a broad spectrum of clinical manifestations, which can be divided into three classes (7): (1) symptoms associated with coexisting congenital abnormalities; (2) those related to abnormal hepatic development, ranging from fatty liver to nodular liver lesions; (3) those resulting from the shunt, including hyperammonemia, PH, hepatopulmonary syndrome.

Abernethy malformation is often observed in association with other congenital anomalies (7). Of the 341 patients, 151 (44.3%) were diagnosed with one or more other congenital anomalies, with congenital heart disease (78/151, 51.7%) being the most common. 29 patients (8.5%) exhibited comorbid AIVC, predominantly in pediatric patients (*P* < 0.05, Table 2). It is hypothesized that this may be associated with the increased likelihood of earlier detection of combinations of these congenital diseases. Our patient is an adult male with a left IVC. While congenital anomalies are more commonly seen in type I as compared to type II (6), our analysis suggests that no significant difference in the incidence of IVC abnormalities between the two types. This could be partly attributable to the inherent relative rarity of IVC anomalies limiting statistical power. A more plausible explanation involves their shared embryological origin. The association of AIVC with Abernethy malformation may be attributed to their close embryological development. Right-sided venous hypoplasia or agenesis affects both the vitelline (portal) and subcardinal (infrahepatic caval vein) venous systems (3). The coincidence of associations in both types also suggests that the two variants are closely related (7). Future studies with detailed phenotyping and larger cohorts are warranted to further elucidate these relationships.

**TABLE 2:** Inferior vena cava anomalies, nodular liver lesions, pulmonary hypertension, and death case in patients with Abernethy malformation.

Clinical features	Number	Type		*P* value	Gender		*P* value	Age		*P* value	Regional distribution		*P* value
		I	II		Male	Female		Child	Adult		Asia	Europe and North America	
Inferior vena cava anomalies	29	14	15	0.833	17	12	0.332	24	5	0.034	14	13	0.758
Nodular liver lesions	124	83	40	<0.001	59	65	0.498	77	47	0.498	54	66	0.027
FNH	58	40	18	0.002	18	40	0.002	43	15	0.086	33	24	0.403
NRH	37	26	10	0.005	23	14	0.117	23	14	0.765	16	18	0.469
HCC	26	20	6	0.004	9	17	0.102	6	20	<0.001	4	22	<0.001
Adenoma	21	15	6	0.044	7	14	0.115	12	9	0.474	5	16	0.006
Pulmonary hypertension	66	31	34	0.660	30	35	0.490	43	23	0.948	35	29	0.999
Death case	20	10	9	0.824	10	10	1.000	15	5	0.325	6	12	0.061

Additionally, Abernethy malformation is often complicated by NLL, which can be divided into benign and malignant according to their pathology. Benign nodules include focal nodular hyperplasia (FNH), nodular regenerative hyperplasia (NRH), adenoma and hemangioma, while malignant nodules include HCC and hepatoblastoma. Of the 341 patients, 124 (36.4%) had combined liver lesions, mainly including FNH (58/124,46.8%), NRH (37/124,29.8%), HCC (26/124,21.0%) and adenoma (21/124,16.9%). Statistically significant differences were observed between Type I and Type II patients in the overall incidence of NLL, as well as in the incidence of FNH, NRH, HCC and adenoma (all *P* < 0.05, Table 2), with these lesions being more commonly seen in Type I. The incidence of FNH also differed significantly between male and female patients (*P* < 0.05, Table 2), with a higher prevalence in females. The incidence of HCC was significantly higher in adult patients compared to pediatric patients (*P* < 0.05, Table 2). Additionally, significant regional differences were observed in the incidence of NLL, HCC, and adenomas among reported cases from Asia and Europe and North America (all *P* < 0.05, Table 2), with a higher prevalence in Europe and North America. Other less common NLL included hepatoblastoma (9 cases), hemangioma (4 cases), and combined hepatocellular carcinoma and cholangiocarcinoma (cHCC-CCA) (1 case). Patients could presented with multiple NLL concurrently. In our patient, MRI revealed multiple NLL, and FNH and solitary necrotic nodule of the liver were considered the most probable diagnoses. Contrast-enhanced CT also showed the multiple enhanced NLL in the arterial phase.

Abnormal oxygen supply appears to be a key driver in causing NLL to form (8). Insufficient portal venous supply results in dilation of the hepatic arteries and increased arterial blood flow, leading to increased oxygen supply. Patients with Abernethy malformation may experience increased exposure of hepatocytes to oxygen free-radicals (9). This may elucidate the observation that NLL are more prevalent in Type I, where the portal vein is entirely absent. Concurrently, shunting results in aberrant hepatotrophic substances composition, including estrogen. Estrogen is clearly associated with the development of FNH (10). Up to three quarters of females with FNH were found to be long-term users of oral contraceptives, suggesting a potential link to its pathogenesis (11). Estrogen receptors have been detected in FNH lesions and surrounding liver parenchyma (12). This may be attributed to the fact that focal nodular hyperplasia is more prevalent in females. The progression of benign NLL complicated by Abernethy malformation to malignancy is a potential concern (13–16). Therefore, such patients should be followed up, and the imaging alterations or elevated tumor markers should prompt consideration of malignant progression, and liver biopsy should be performed if necessary. Following the closure of the shunt, the NLL may resolve completely or partially (10).

Common non-hepatic complications of Abernethy malformation include hyperammonemia (108/341, 31.7%), PH (66/341, 19.4%), hepatopulmonary syndrome (58/341, 17.0%), hepatic encephalopathy (HE) (51/341, 15.0%), gastrointestinal hemorrhage (34/341, 10.0%), developmental delay (33/341, 9.7%) and intellectual disability (23/341, 6.7%). Less common complications include hypoglycemia, glomerular disease, hyperandrogenemia, osteoporosis and hypothyroxinemia. PH is a common pulmonary complication. The pathophysiology of PH in the setting of Abernethy malformation is considered a portopulmonary hypertension, although portal hypertension is not a feature of Abernethy malformation (17). The portosystemic shunt diverts portal blood flow into the systemic circulation, enabling the bypass of the liver and the direct entry of vascular mediators, proinflammatory cytokines, proangiogenic factors, and bacterial endotoxins into the pulmonary circulation from the mesenteric circulation. They can inflict damage upon the vascular pulmonary endothelium and stimulate endothelial cell proliferation, smooth muscle hypertrophy, and *in situ* thrombosis. Furthermore, blood clots from the mesenteric circulation can enter the pulmonary circulation via the portosystemic shunt. High cardiac output also promotes hypertrophy, proliferation and vasoconstriction of pulmonary endothelial cells. The aforementioned factors all contribute to the development of PH (18).

The patient presented with dilated portal vein and splenomegaly. We speculate that this patient may have a congenital hypoplastic portal vein system. Aligning with the report by Yao X et al. (19), because of the thin portal vein branch, the blood flow returning to the liver was blocked, forming regional portal hypertension and splenomegaly. The dilatation main portal vein was considered compensatory dilatation. It is a rare case of Abernethy malformation manifesting both shunt-mediated pulmonary hypertension and regional portal hypertension.

In addition, cardiopulmonary causes are the main causes of death in patients with Abernethy malformation. A total of 20 deaths were reported in 341 cases. Of these, 8 were due to cardiopulmonary causes, 6 of which were combined with PH (20–24). 2 patients died as a result of coronary artery disease caused by compression of the coronary arteries following pulmonary vasodilatation in PH (21, 24). 1 patient died as a result of pulmonary heart disease caused by PH (20). 1 patient, due to prolonged absence of intervention for PH, surgical treatment for CEPS had to be performed when treatment for PH was initiated but the target hemodynamic profile was not achieved, resulting in the patient’s death in the early postoperative period (25). It is therefore recommended that active intervention be performed in cases of Abernethy malformation with PH, in order to avoid further progression and to be alert to the poor prognosis that may result. Optimal management of patients with PH requires a multidisciplinary approach to determine the timing of shunt closure, medical PAH therapy and the indication for lung, liver, or combined transplantation in the most severe cases (26). Life-threatening visceral aneurysms may also occur, and the awareness of this unusual entity is crucial for the prevention and close monitoring of possible complications, such as abdominal hemorrhage (27). For patients with aneurysms, attention should be paid to the treatment strategy to prevent a potentially fatal aneurysm rupture (28).

## Conclusion

We present the case of a 21-year-old male patient with Abernethy malformation type II with a left IVC, PH and NLL. Abernethy malformation presents with a range of clinical manifestations. It should be considered in patients with unexplained PH in clinical practice. Although rare, it may cause portal hypertension. Imaging examinations such as CTA can assist in confirming the diagnosis and detecting concomitant diseases, including nodular hepatic lesions and AIVC. It is crucial to diagnose and treat Abernethy malformation promptly, as well as to screen and prevent related diseases for a better prognosis.

## Data Availability

The original contributions presented in this study are included in this article/Supplementary material, further inquiries can be directed to the corresponding author.
