# Supplementary material for: Pulmonary hypertension secondary to Abernethy malformation with left inferior vena cava: a case report and literature review
Source: Front Med (Lausanne). 2025 Aug 22;12:1604853. doi: 10.3389/fmed.2025.1604853 (PMC12411206; doi:10.3389/fmed.2025.1604853)
Supplement: Supplementary file 1 [file Data_Sheet_1.docx]

Supplementary material

**S1**

Echocardiography revealed significant abnormalities primarily involving the right heart and pulmonary circulation.

The right atrium was severely dilated, with dimensions measuring 54 mm*46 mm and an area of 23 cm². The right ventricle (RV) was markedly enlarged, evidenced by an RV basal diameter of 44 mm and a mid-cavity diameter of 34 mm. Further indicators of significant RV pressure and volume overload included flattening of the interventricular septum leading to a "D-shaped" left ventricle (LV) in systole (eccentricity index 1.43), dilation of the main pulmonary artery (31 mm), and foreshortened RV outflow tract acceleration time (77 ms). Assessment of RV function yielded mixed indices: systolic longitudinal motion measured by tricuspid annular plane systolic excursion (TAPSE=24 mm) and peak systolic tissue Doppler velocity at the lateral tricuspid annulus (s'=19 cm/s) were within normal limits, suggesting preserved longitudinal systolic function. However, the right ventricular index of myocardial performance (RIMP or Tei index) measured by tissue Doppler was significantly elevated at 0.89, indicating overall RV dysfunction. Pulmonary artery systolic pressure (PASP) of about 82 mmHg estimated by the tricuspid regurgitation pressure gradient method.

Left ventricular size was normal (37 mm), with preserved global systolic function (left ventricular ejection fraction, LVEF 60%) and normal wall thickness (interventricular septum 9 mm, posterior wall 11 mm). Left atrial size was normal (25 mm). Mild mitral regurgitation was present. Atrial arrangement (situs solitus) and atrioventricular and ventriculo-arterial connections were normal, with no evidence of significant shunts.

**S2**

Right heart catheterization showed: pulmonary arterial pressure (77/22/42mmHg), pulmonary arterial wedge pressure (8/1/3mmHg), pulmonary vascular resistance (4.88 Wood units), right atrial pressure (7/0/4mmHg), cardiac output (8.0 L/min), and cardiac index (4.57 L/min/m²).

**S3 295 Studies included in review**

1. Guo F, Ding D, Zhang G. Congenital extrahepatic portosystemic shunt in a two-month-old infant. Asian J Surg. Published online September 12, 2024:S1015-9584(24)01959-6. doi:10.1016/j.asjsur.2024.08.219

2. Pan J, Pan H, Kong L, Li X. A case of Type II Abernethy malformation with multiple intrahepatic nodules. Asian Journal of Surgery. Published online September 10, 2024. doi:10.1016/j.asjsur.2024.09.030

3. Shakya S, Chataut D, Paudel S, Gajurel RM. Transcatheter closure of congenital extrahepatic portosystemic shunt to right atrium causing severe pulmonary hypertension in a child: A case report. Progress in Pediatric Cardiology. 2024;74:101729. doi:10.1016/j.ppedcard.2024.101729

4. Liu X, Ye W, Nie CC, Wei A. A case of Abernethy malformation type I combined with hepatoblastoma. Rev Esp Enferm Dig. Published online July 11, 2024. doi:10.17235/reed.2024.10605/2024

5. Sharma P, Panghal S, Sharma S, Sharma N, Lakhani N, Meena B. Abernethy Malformation: A Rare Case Report. JK Science: Journal of Medical Education & Research. 2024;26(3):187-189.

6. Jacob A, DaCosta S, Miller M, Newman J, Davis J. AN OVERLOOKED CAUSE OF RECURRENT HEPATIC ENCEPHALOPATHY: A RARE CASE OF SUPERIOR MESENTERIC TO RIGHT RENAL VEIN PORTOSYSTEMIC SHUNT. Gastrointestinal Endoscopy. 2024;99(6):AB76-AB77. doi:10.1016/j.gie.2024.04.799

7. Tom CK, Ter-Oganesyan R, Shefali C, et al. Benign to Malignant Hepatic Lesion Transformation in Abernethy Malformation. ACG Case Rep J. 2024;11(4):e01307. doi:10.14309/crj.0000000000001307

8. Xu Z, Yin X. Unraveling the mystery: congenital absence of the portal vein and its cardiac implications. Rev Esp Enferm Dig. Published online March 7, 2024. doi:10.17235/reed.2024.10323/2024

9. Ye Z, Li Z. Cinematic Rendering of Abernethy Malformation with Abdominal Aneurysms. Radiology. 2024;310(2):e232202. doi:10.1148/radiol.232202

10. Wadile S, Banpurkar A, Kulkarni S. Trans-catheter closure of an unusual type of Abernethy malformation in a child presenting with severe pulmonary hypertension. Cardiol Young. 2024;34(2):436-438. doi:10.1017/S1047951123004006

11. Bhatte S, Cahill AM, Dunn M, Foran A, Perez A, Acord MR. Endovascular closure of a congenital extrahepatic portosystemic shunt for the treatment of hepatopulmonary syndrome in an infant. Pediatr Radiol. 2024;54(2):357-361. doi:10.1007/s00247-023-05837-w

12. AlMheiri M, Mrayyan HB, Krishnamurthy B, Dsouza AP. Congenital Extrahepatic Portosystemic Shunt Complicated by the Development of Hepatoblastoma: A Case Report and Review of Literature. Cureus. 2024;16(2):e54508. doi:10.7759/cureus.54508

13. Miklashevich IM, Potrokhova EA, Morozov DA, Isaeva YS. Pulmonary arterial hypertension associated with type II Abernethy malformation in an adolescent: a case report. Cardiovascular Therapy and Prevention. 2024;23(2):3754. doi:10.15829/1728-8800-2024-3754

14. Shastri A, De A, Chaluvashetty SB, Duseja AK. Unraveling a rare culprit of hepatic encephalopathy in adulthood. Journal of Clinical and Experimental Hepatology. 2024;14. doi:10.1016/j.jceh.2024.102326

15. Oleti RT, Ardhanari R. A case report of abernethy type II malformation. HPB. 2024;26:S275. doi:10.1016/j.hpb.2024.03.553

16. Yan J, Yin J, Zhang D, Lv C, Pang W, Chen Y. An unusual interventional approach to treat Type 2 Abernethy malformation in children: two case reports. Gastroenterol Rep (Oxf). 2024;12(1):goad075. doi:10.1093/gastro/goad075

17. Bade R, Peters NJ, Mathew JL, Sen IM, Mahajan JK. Surgically Correctable Central Cyanosis: Congenital Extrahepatic Portosystemic Shunt in a Child. J Indian Assoc Pediatr Surg. 2024;29(4):376-378. doi:10.4103/jiaps.jiaps_1_24

18. Singh T, Rose S, Kalra N, Duseja A. Regenerative nodules as harbinger of Abernethy malformation. BMJ Case Rep. 2023;16(12):e258634. doi:10.1136/bcr-2023-258634

19. Zhang LJ, Liu XY, Chen TF, Xu ZY, Yin HJ. Type II Abernethy malformation with cystic fibrosis in a 12-year-old girl: A case report. World J Clin Cases. 2023;11(32):7865-7871. doi:10.12998/wjcc.v11.i32.7865

20. Zhang D, Wu X, Yang Z, et al. Successful Liver Transplantation From a Deceased Donor With Congenital Extrahepatic Portosystemic Shunt: A Case Report. Transplantation Proceedings. 2023;55(8):1943-1945. doi:10.1016/j.transproceed.2023.07.014

21. Malik A, Patel ME, Ganger D, Hohlastos E, Riaz A. Percutaneous sequential closure of an Abernethy malformation: A case report. Radiology Case Reports. 2023;18(9):3135-3139. doi:10.1016/j.radcr.2023.06.019

22. Yao X, Liu Y, Yu LD, Qin JP. Rare portal hypertension caused by Abernethy malformation (Type IIC): A case report. World J Radiol. 2023;15(8):250-255. doi:10.4329/wjr.v15.i8.250

23. Tamiru R, Hailemariam T, Wakjira E, Hailu SS. Congenital extrahepatic portosystemic shunt masquerading as chronic portal vein thrombosis: A case report. International Journal of Surgery Case Reports. 2023;109:108553. doi:10.1016/j.ijscr.2023.108553

24. Jiao J, Finberg KE, Jain D, Morotti R. Hepatocellular Adenoma: Report of 2 Cases That Highlight the Relevance of Phenotype-Genotype Correlation in the Pediatric Population. Pediatric and developmental pathology : the official journal of the Society for Pediatric Pathology and the Paediatric Pathology Society. Published online June 19, 2023:10935266231175426. doi:10.1177/10935266231175426

25. Pawal S, Arkar R. A Rare Cause of Recurrent Encephalopathy in a Septuagenarian: A Case Report. Cureus. 2023;15(6):e41015. doi:10.7759/cureus.41015

26. Brun MM, Cardoso MR, Borim BC, Marchi CH, Croti UA. Abernethy Malformation: Possible Diagnosis for Patients with Congenital Heart Disease and Persistent Cyanosis. Brazilian Journal of Cardiovascular Surgery. 2023;38(2):300-304. doi:10.21470/1678-9741-2022-0110

27. Morneault K, Mathews A, Sharma P, Beasley G. Congenital Portosystemic Shunt Presenting As Hyperammonemia Following Fontan Operation. JPGN reports. 2023;4(1):e282. doi:10.1097/pg9.0000000000000282

28. Agarwal R, Prasad D, Chauhan G, Verma A. Abernethy Malformation Masquerading as Congenital Heart Disease: A Boy With Cyanosis, Clubbing, and Hypoxia. Cureus. 2023;15(1):e33519. doi:10.7759/cureus.33519

29. Yalcin A, Ceylan M, Cakir M, Ceylan O, Yilmaz A. Deep brain stimulation for the abernethy malformation related tremor. Clinical Neurology and Neurosurgery. 2023;224. doi:10.1016/j.clineuro.2022.107554

30. Xu L, Zhang H, Liu G, Li Y, Li D, Ma N. Abernethy malformation with unusual cardiac malformation: Case report and literature review. Echocardiography. 2023;40(1):57-60. doi:10.1111/echo.15504

31. Hailemariam T, Demilew E, Manyazewal T, Sisay S. Congenital extrahepatic Portosystemic shunt with hypoplasia of the intrahepatic inferior vena cava: A rare case report. Radiology Case Reports. 2023;18(8):2750-2753. doi:10.1016/j.radcr.2023.05.042

32. Gao YY, Tang Q, Liu YC, et al. Unexplained hyperammonemia and encephalopathy in the emergency department: Abernethy malformation in elderly patients. World Journal of Emergency Medicine. 2023;14(1):69-71. doi:10.5847/wjem.j.1920-8642.2023.008

33. Fukuda S, Hasegawa N, Mori K, et al. Shrinkage of Enlarged Hepatic Nodules by an Embolizing Congenital Extrahepatic Portosystemic Shunt. Intern Med. Published online 2023:2267-23. doi:10.2169/internalmedicine.2267-23

34. Beal EW, Foley K, Washburn K, Tsung A. Congenital Absence of the Portal Vein. American Surgeon. 2023;89(4):1031-1033. doi:10.1177/0003134820960064

35. 李良辉, 胡加银. 超声联合CT诊断Abernethy畸形合并单侧下肢静脉曲张1例. 中国临床医学影像杂志. 2023;34(6):449-450.

36. Tessier S, Ido F, Zanders T, Longo S, Nanda S. Congenital Porto-Azygous Shunt (Abernethy Malformation Type II) in an Elderly Patient: A Too-Often-Forgotten Occult Abnormality. Cureus. 2022;14(4):e24460. doi:10.7759/cureus.24460

37. Ghasemi-Rad M, Smuclovisky E, Cleveland H, Hernandez JA. Endovascular treatment of a portosystemic shunt presenting with hypoglycemia; case presentation and review of literature. Clinical Imaging. 2022;83:131-137. doi:10.1016/j.clinimag.2021.12.020

38. Bahadori A, Kuhlmann B, Debray D, et al. Presentation of Congenital Portosystemic Shunts in Children. Children (Basel, Switzerland). 2022;9(2). doi:10.3390/children9020243

39. Shakya S, Saxena A, Ramakrishnan S. Transcatheter closure of Abernethy malformation associated with interrupted inferior caval vein and other systemic venous anomalies. Cardiology in the Young. 2022;32(2):337-339. doi:10.1017/s1047951121002900

40. Lin X qin, Rao J yi, Xiang Y fei, et al. Case Report: A Rare Syncope Case Caused by Abernethy II and a Review of the Literature. Frontiers in cardiovascular medicine. 2022;8. doi:10.3389/fcvm.2021.784739

41. Zhu B, Tian H, Song FJ, et al. Abernethy malformation associated with COACH syndrome in a patient with TMEM67 mutation: a case report. Zhonghua nei ke za zhi. 2022;61(9):1052-1055. doi:10.3760/cma.j.cn112138-20220107-00019

42. Zhen L, Han J, Li DZ. Congenital extrahepatic portosystemic shunt: An unusual feature in cardio-facio-cutaneous syndrome. European Journal of Obstetrics and Gynecology and Reproductive Biology. 2022;273:107-108. doi:10.1016/j.ejogrb.2022.04.022

43. Yamada K, Matsukuma S, Tokumitsu Y, Shindo Y, Ikeda Y, Nagano H. Surgical shunt ligation for a congenital extrahepatic portosystemic shunt with pulmonary hypertension: A case report. International Journal of Surgery Case Reports. 2022;93. doi:10.1016/j.ijscr.2022.107024

44. Wu X, Gu W, Lin Y, Ye L. A rare presentation of type II Abernethy malformation and nephrotic syndrome: Case report and review. Open life sciences. 2022;17(1):794-799. doi:10.1515/biol-2022-0086

45. Sieverding L, Hofbeck M, Michel J, et al. A new variant of Abernethy malformation treated by transhepatic interventional closure: a case report. BMC Gastroenterology. 2022;22(1). doi:10.1186/s12876-022-02123-1

46. Saini T, Singh RK, Prasad U, Agrawal L. Abernethy Malformation with Liver SOL. Indian Journal of Surgery. Published online 2022. doi:10.1007/s12262-022-03615-w

47. Rabiee P, Saedi S. Multiple congenital visceral abnormalities as a rare cause of pulmonary arterial hypertension. Egyptian Heart Journal. 2022;74(1). doi:10.1186/s43044-022-00273-x

48. Kruszyna T, Rogala J, Jędrychowski T, et al. Kidney Transplantation in Abernethy Malformation: A Case Report. Transplantation Proceedings. 2022;54(4):1155-1157. doi:10.1016/j.transproceed.2022.02.013

49. kayedi M, Kian B, Teimouri A. A rare Abernethy Ib malformation was initially misdiagnosed as chronic portal vein thrombosis in a 27-year-old female. Radiology Case Reports. 2022;17(10):3551-3555. doi:10.1016/j.radcr.2022.06.093

50. Ji L, Ji Z, Xiang D, Qin Y, Yang S. Case report: Rare abernethy malformation with hepatopulmonary syndrome in a pediatric patient. Frontiers in Pediatrics. 2022;10. doi:10.3389/fped.2022.856611

51. Hlavata T, Kaldararova M, Klauco F, Drangova E, Reptova A, Simkova I. Congenital Absence of the Portal Vein as a Rare Cause of Portopulmonary Hypertension-A Case Study Series. Medicina (Kaunas, Lithuania). 2022;58(10). doi:10.3390/medicina58101484

52. Harlander M, Badovinac M, Markoska F, et al. Case report: Congenital extrahepatic portocaval shunt presenting as pulmonary arterial hypertension in a pregnant patient. Pulmonary Circulation. 2022;12(1). doi:10.1002/pul2.12008

53. Goncalves I, Barros D, Araujo M, Machado AI, Oliveira C, Pinto L. Type II Abernethy Malformation: A Rare Cause of Hepatic Encephalopathy in Adulthood. European Journal of Case Reports in Internal Medicine. 2022;9(1):003145-003145. doi:10.12890/2022_003145

54. El-Medany AY, Rego G, Williams M, Lyen S, Turner M. Multimodality imaging of Abernethy malformation. Echocardiography. 2022;39(3):524-527. doi:10.1111/echo.15324

55. Bombardier B, Alli A, Rohr A, Collins Z, Raval K. A case of two shunts in the endovascular treatment of type II Abernethy syndrome. CVIR Endovascular. 2022;5(1). doi:10.1186/s42155-021-00279-7

56. Abdullah L, Hussein L, Al Houri HN, Khouri L. Abernethy malformation (Type 1B) presenting in a 6-year-old boy with hematochezia and hematuria: A case report. Radiology Case Reports. 2022;17(9):3318-3320. doi:10.1016/j.radcr.2022.06.045

57. Andrade G, Facas J, Marques P, Monica AN, Donato P. Congenital extrahepatic portosystemic shunt type II occluded with cardiac closure device. Radiology Case Reports. 2021;16(12):3802-3806. doi:10.1016/j.radcr.2021.09.020

58. Sasikumar D, Valakkada J, Kramadhari H, Ayyappan A, Krishnamoorthy KM. Novel transcatheter treatment for staged closure of Abernethy malformation with portal hypoplasia. Annals of Pediatric Cardiology. 2021;14(3):419-421. doi:10.4103/apc.APC_189_20

59. Namgoong JM, Hwang S, Park GC, Kwon H, Kim KM, Oh SH. Living donor liver transplantation in a pediatric patient with congenital absence of the portal vein. Annals of hepato-biliary-pancreatic surgery. 2021;25(3):401-407. doi:10.14701/ahbps.2021.25.3.401

60. Loureiro P, Georgiev S, Ewert P, et al. Successful percutaneous treatment with the Konar MF™-VSD Occluder in an infant with Abernethy syndrome-case report. Cardiovascular Diagnosis and Therapy. 2021;11(2):631-636. doi:10.21037/cdt-20-380

61. Namgoong JM, Hwang S, Kim DY, et al. Pediatric liver transplantation using a hepatitis B surface antigen-positive donor liver graft for congenital absence of the portal vein. Korean journal of transplantation. 2021;35(1):59-65. doi:10.4285/kjt.20.0038

62. Zhao X, Zheng J, Li P, Li Y. Congenital extrahepatic portosystemic shunt in adults: A report of three cases. Journal of Clinical Hepatology. 2021;37(4):910-913. doi:10.3969/j.issn.1001-5256.2021.04.038

63. Zeng P, Qu ZF. A case of interventional treatment of Abernethy malformation with pulmonary hypertension. Journal of Clinical Pediatric Surgery. 2021;20(5):495-497. doi:10.12260/lcxewkzz.2021.05.021

64. Yao X, Liao J. Multidetector CT findings of Abernethy malformation typeb in child with liver transplantation. Chinese Journal of Radiology (China). 2021;55(1):76-77. doi:10.3760/cma.j.cn112149-20200113-00042

65. Tanaka H, Saijo Y, Tomonari T, et al. An adult case of congenital extrahepatic portosystemic shunt successfully treated with balloon-occluded retrograde transvenous obliteration. Internal Medicine. 2021;60(12):1839-1845. doi:10.2169/internalmedicine.5914-20

66. Rout A, Chongthammakun V, Siegel YJ, Mendoza D, Damluji AA. Abernethy malformation with massively dilated main pulmonary artery manifesting as acute myocardial infarction. Journal of Cardiovascular Imaging. 2021;29(4):379-381. doi:10.4250/JCVI.2021.0011

67. Ringle ML, Loomba R, Dykes JC, Khan D, Schidlow D, Wernovsky G. The multisystem nature of isomerism: Left isomerism complicated by Abernethy malformation and portopulmonary hypertension. Cardiology in the Young. 2021;31(4):532-540. doi:10.1017/S1047951121000809

68. Raju SN, Pandey NN, Singh SP, et al. Successful Embolization of Type II Congenital Extrahepatic Portosystemic Shunt Using Amplatzer Septal Occluder and Amplatzer Vascular Plug II. Journal of Vascular and Interventional Radiology. 2021;32(8):1236-1237. doi:10.1016/j.jvir.2021.05.022

69. Nandan R, Jana M, Yadav DK, Goel P. Cystic biliary atresia with congenital absence of portal vein. BMJ Case Reports. 2021;14(1). doi:10.1136/bcr-2020-239205

70. Nakajima H, Kodo K. A 3-year-old boy with congenital absence of the portal vein presenting fasting hypoglycemia and postprandial hyperglycemia with hyperinsulinemia. Journal of Pediatric Surgery Case Reports. 2021;75. doi:10.1016/j.epsc.2021.102052

71. Mingkun L, Xinghai C, Yifan F, Dianming W, Long L. Type II Abernethy malformation combined with focal nodular hyperplasia of the liver: a case report and literature review. Chinese Journal of Hepatobiliary Surgery. 2021;27(12):941-942. doi:10.3760/cma.j.cn113884-20210330-00116

72. Migdał A, Koleśnik A, Żuk M, Brzezińska-Rajszys G. Pulmonary arterial hypertension secondary to congenital extrahepatic portosystemic shunt - a serious complication of a rare disease. Kardiologia Polska. 2021;79(11):1288-1289. doi:10.33963/KP.a2021.0096

73. Liu Z, Niu C, Zhang J, Shen G. Successful embolization for type Ⅱ Abernethy malformation: one case report. Chinese Journal of Pediatric Surgery. 2021;42(11):1033-1035. doi:10.3760/cma.j.cn421158-20200526-00370

74. Laverdure N, Lallier M, Dubois J, Paganelli M. Congenital absence of the portal vein: Define the portosystemic shunt, avoid liver transplantation. Canadian Liver Journal. 2021;4(3):322-327. doi:10.3138/canlivj-2020-0011

75. He X, Zhu Y, Fu H, et al. Case Report: Membranoproliferative Glomerulonephritis, a Rare Clinical Manifestation of Abernethy Malformation Type II. Frontiers in Pediatrics. 2021;9. doi:10.3389/fped.2021.647364

76. Figueras-Coll M, Sabaté-Rotés A, Iglesias-Serrano I, Peñas-Aguilera A, Pérez-Lafuente M. Abernethy Malformation: An Unusual Extrathoracic Cause of Chronic Hypoxemia in Pediatrics. Archivos de Bronconeumologia. 2021;57(12):782-784. doi:10.1016/j.arbres.2021.02.005

77. Facas J, Cruz M, Costa JF, Agostinho A, Donato P. Multistage closure of a congenital extrahepatic portosystemic shunt. CVIR Endovascular. 2021;4(1). doi:10.1186/s42155-021-00267-x

78. Claesen E, Berge SVD, Havinga E. Abernethy Malformation Type 1b. Journal of the Belgian Society of Radiology. 2021;105(1). doi:10.5334/JBSR.2431

79. Berg S, Araujo Perez T. Giant HNF 1α-Inactivated Liver Adenoma in a Nine-Year-Old Boy with Abernethy Malformation Type II. RoFo Fortschritte auf dem Gebiet der Rontgenstrahlen und der Bildgebenden Verfahren. 2021;193(1):84-87. doi:10.1055/a-1207-0935

80. Sasikumar D, Ayyappan A, Valakkada J, Krishnamoorthy KM. Diagnosing pulmonary arteriovenous malformations in the presence of atrial septal defect and anomalous pulmonary venous drainage-An imaging challenge. Echocardiography. 2020;37(9):1492-1494. doi:10.1111/echo.14817

81. Cruz M, Facas J, Donato P. ARP Case Report Nº 19: Congenital Extrahepatic Portosystemic Shunt (Abernethy Malformation). Acta Radiológica Portuguesa. 2020;32(2):15-17. doi:10.25748/arp.20957

82. Li P, Hidaka M, Hamada T, et al. Living donor liver transplantation for congenital absence of portal vein in portal venous reconstruction with a great saphenous vein graft. Surgical case reports. 2020;6(1):153. doi:10.1186/s40792-020-00916-8

83. Nam HD. Living-donor liver transplantation for Abernethy malformation - case report and review of literature. Annals of hepato-biliary-pancreatic surgery. 2020;24(2):203-208. doi:10.14701/ahbps.2020.24.2.203

84. Thanh Le V, Dung Dao D, Nam Hoang D, Quang Vu V. The first report of living donor liver transplantation for abernethy malformation (congenital absence of the portal vein) in Vietnam. Journal of Pediatric Surgery Case Reports. 2020;55. doi:10.1016/j.epsc.2020.101419

85. Zhou M, Zhang J, Luo L, et al. Surgical Ligation for the Treatment of an Unusual Presentation of Type II Abernethy Malformation. Annals of Vascular Surgery. 2020;65:285.e1-285.e5. doi:10.1016/j.avsg.2019.10.094

86. Yam MKH, Cheung KO, Sim SW, Lee PSF. An adolescent girl in Hong Kong with type Ib Abernethy malformation complicated by multiple focal nodular hyperplasia. Radiology Case Reports. 2020;15(11):2477-2481. doi:10.1016/j.radcr.2020.09.016

87. Thirapattaraphan C, Treepongkaruna S, Ruangwattanapaisarn N, Sae-Guay S. Congenital extrahepatic portosystemic shunt (Abernethy malformation) treated with surgical shunt ligation: A case report and literature review. International Journal of Surgery Case Reports. 2020;66:4-7. doi:10.1016/j.ijscr.2019.11.014

88. Taydas O, Danisan G, Ogul H, Kantarci M. A rare cause of congenital portosystemic shunt: type 2 Abernethy malformation. Folia morphologica. 2020;79(1):172-175. doi:10.5603/FM.a2019.0063

89. Stepffer C, Marques A, Barbosa JD, Ferrario S, Haag D. Pulmonary Arterial Hypertension in a Patient with a Portosystemic Shunt: Diagnostic Challenge. CASE. 2020;4(2):93-96. doi:10.1016/j.case.2019.08.007

90. Sonawane B, Sivakumar K. Abernethy malformation draining above the diaphragm to the coronary sinus and its embryological explanations. Cardiology in the Young. 2020;30(12):1930-1932. doi:10.1017/S1047951120003571

91. Peček J, Fister P, Homan M. Abernethy syndrome in Slovenian children: Five case reports and review of literature. World Journal of Gastroenterology. 2020;26(37):5731-5744. doi:10.3748/wjg.v26.i37.5731

92. Matsumoto N, Matsusaki T, Hiroi K, et al. Pediatric Living Donor Liver Transplantation for Congenital Absence of the Portal Vein With Pulmonary Hypertension: A Case Report. Transplantation Proceedings. 2020;52(2):630-633. doi:10.1016/j.transproceed.2019.11.032

93. Liu Y, Wang WW, Liu Y, Liu Z, Chen WY. Abernethy malformation with IgA nephropathy in a child. Zhonghua er ke za zhi = Chinese journal of pediatrics. 2020;58(7):607-608. doi:10.3760/cma.j.cn112140-20191211-00801

94. Lin Y, Li X, Li S, Ba H, Wang H, Zhu L. Treatment Option for Abernethy Malformation-Two Cases Report and Review of the Literature. Frontiers in Pediatrics. 2020;8:497447. doi:10.3389/fped.2020.497447

95. Jaklitsch M, Sobral M, Carvalho AM, Marques HP. Abernethy malformation and hepatocellular carcinoma: A serious consequence of a rare disease. BMJ Case Reports. 2020;13(1). doi:10.1136/bcr-2019-231843

96. Glonnegger H, Schulze M, Kathemann S, et al. Case Report: Hepatic Adenoma in a Child With a Congenital Extrahepatic Portosystemic Shunt. Frontiers in Pediatrics. 2020;8. doi:10.3389/fped.2020.00501

97. Das JN, Ghosh S, Pathak NL, Chattopadhyay A. Atrial septal defect with unexplained cyanosis and surprising turn of events-A case report. IHJ Cardiovascular Case Reports (CVCR). 2020;4(2):83-86. doi:10.1016/j.ihjccr.2020.07.002

98. Chiang J, Chiu HK, Moriarty JM, McWilliams JP. Hyperandrogenism and malignant degeneration of hepatic adenomas in the setting of Abernethy malformation. Radiology Case Reports. 2020;15(12):2701-2705. doi:10.1016/j.radcr.2020.10.026

99. Bessho H, Tanaka S, Ishihara A, et al. Hepatocellular carcinoma in an adult patient with congenital absence of the portal vein type II: A case report. JGH Open. 2020;4(4):766-768. doi:10.1002/jgh3.12312

100. Kaya MN, Toprak O, Turel S, Ergun U, Arslan TY. Congenital Extrahepatic Portosystemic Shunt: Abernethy Malformation Type 2. European Journal of Therapeutics. 2019;25(3):230-232. doi:10.5152/EurJTher.2019.600

101. Xiang W, Wang H, Si ZZ, Chen GS, Wang GW, Li T. Type I congenital extrahepatic portosystemic shunt treated by orthotopic liver transplantation: A case report. World journal of clinical cases. 2019;7(7):903-907. doi:10.12998/wjcc.v7.i7.903

102. Zheng Y, Peng Y, Zhang S, Li L, Peng Y, Yin Q. Capillary Malformation-Arteriovenous Malformation Combined Alagille Syndrome in a Patient With Double Gene Variations of RASA1 and NOTCH2. Frontiers in Genetics. 2019;10:1088. doi:10.3389/fgene.2019.01088

103. Xie L, Li Y, Jiang X, Zhao J, Xiao T. A 10-year-old boy with dyspnea and hypoxia: Abernathy malformation masquerading as pulmonary arteriovenous fistula. BMC Pediatrics. 2019;19(1). doi:10.1186/s12887-019-1422-x

104. Sakaki S, Hayashi T, Ono H. Pulmonary arteriovenous malformations in a patient with single ventricle and polysplenia syndrome. BMJ Case Reports. 2019;12(7). doi:10.1136/bcr-2019-229491

105. Romano R, Grasso F, Gallo V, et al. A case of incontinentia pigmenti associated with congenital absence of portal vein system and nodular regenerative hyperplasia. British Journal of Dermatology. 2019;180(3):674-675. doi:10.1111/bjd.17319

106. Ponziani FR, Faccia M, Zocco MA, et al. Congenital extrahepatic portosystemic shunt: description of four cases and review of the literature. Journal of Ultrasound. 2019;22(3):349-358. doi:10.1007/s40477-018-0329-y

107. Păcurar D, Dijmărescu I, Dijmărescu AD, Romaşcanu M, Becheanu CA. A case report on an incidental discovery of congenital portosystemic shunt. Medicine. 2019;98(31):e16679. doi:10.1097/MD.0000000000016679

108. Movilla P, Ohliger M, Wood S. Prepared for the Unexpected: Accessory Ovaries with Abernethy Malformation. Journal of Minimally Invasive Gynecology. 2019;26(5):977-980. doi:10.1016/j.jmig.2018.12.005

109. Mathai SV, Kondray V, Salloum E, Kukreja K, Tavri S. Role of interventional radiology in the diagnosis and management of congenital extrahepatic portosystemic shunts: Two case reports. Indian Journal of Radiology and Imaging. 2019;29(2):219-222. doi:10.4103/ijri.IJRI_461_18

110. Lux D, Naito A, Harikrishnan S. Congenital extrahepatic portosystemic shunt with progressive myelopathy and encephalopathy. Practical neurology. 2019;19(4):368-371. doi:10.1136/practneurol-2018-002111

111. Lin KY, Chen H, Yu L. Pulmonary arterial hypertension caused by congenital extrahepatic portocaval shunt: A case report. BMC Cardiovascular Disorders. 2019;19(1). doi:10.1186/s12872-019-1124-1

112. Kemp SR. Sonography Aids in Diagnosis of Congenital Absence of Portal Vein in Patient With Marfan Syndrome: A Case Study. Journal of Diagnostic Medical Sonography. 2019;35(4):327-333. doi:10.1177/8756479319848747

113. Ding PX, Han XW, Liu C. Type II abernethy malformation in a patient with primary budd-chiari syndrome. Annals of Hepatology. 2019;18(1):246-249. doi:10.5604/01.3001.0012.7933

114. Azad S, Arya A, Sitaraman R, Garg A. Abernethy malformation: Our experience from a tertiary cardiac care center and review of literature. Annals of Pediatric Cardiology. 2019;12(3):240-247. doi:10.4103/apc.APC_185_18

115. Yangın-Ergon E, Ermis N, Colak R, et al. Abernethy Malformation Type 2 and Biliary Atresia Coexistence: A Rare Cause of Infantile Liver Transplant. Euroasian journal of hepato-gastroenterology. 2018;8(2):163-166. doi:10.5005/jp-journals-10018-1283

116. Zhang B, Wu KT, Li L, Lai HY. Catheter Embolization of Type II Congenital Extrahepatic Portosystemic Shunt with Hematochezia: A Case Series and Review of the Literature. CardioVascular and Interventional Radiology. 2018;41(7):1121-1127. doi:10.1007/s00270-018-1972-6

117. Virdis M, Monteleone M, Sposito C, Cascella T, Pellegrinelli A, Mazzaferro V. Hepatocellular Carcinoma in Abernethy Malformation: A Rare Occurrence of Congenital Complete Portosystemic Shunt. Journal of Vascular and Interventional Radiology. 2018;29(12):1775-1778. doi:10.1016/j.jvir.2018.07.014

118. Suárez-Sánchez A, Solar-García L, García-Bernardo C, Miyar-de-León A. Lower gastrointestinal bleeding as a form of presentation in an adult case of Abernethy syndrome. Revista Espanola de Enfermedades Digestivas. 2018;110(10):667-668. doi:10.17235/reed.2018.5615/2018

119. Soota K, Klair JS, LaBrecque D. Confusion for Fifteen Years: A Case of Abernethy Malformation. Clinical Gastroenterology and Hepatology. 2018;16(10):A50. doi:10.1016/j.cgh.2018.01.003

120. Sheth R, Sivakumar K. The Abernethy malformation with inferior caval vein hypoplasia: A tailored technique for transcatheter closure and an insight into embryological perspective. Cardiology in the Young. 2018;28(9):1169-1171. doi:10.1017/S1047951118000884

121. Sharma S, Bobhate PR, Sable S, et al. Abernethy malformation: Single-center experience from India with review of literature. Indian Journal of Gastroenterology. 2018;37(4):359-364. doi:10.1007/s12664-018-0884-3

122. Sasikumar D, Sasidharan B, Ayyappan A. Haemodynamic consequences following closure of an Abernethy malformation in a patient following a total cavopulmonary shunt. Cardiology in the Young. 2018;28(5):768-770. doi:10.1017/S1047951118000185

123. Mohtashami A, Kiat A, Cross J, Simon R, Curtin A. Catastrophic intraoperative bleeding due to congenital extrahepatic porto-systemic shunt anomaly: A surgical case report of two rare anomalies. International Journal of Surgery Case Reports. 2018;44:161-165. doi:10.1016/j.ijscr.2018.02.034

124. Mesquita RD, Sousa M, Vilaverde F, Cardoso R. Abernethy malformation: beware in cases of unexplained hepatic encephalopathy in adults-case report and review of the relevant literature. BJR case reports. 2018;4(2):20170054. doi:10.1259/bjrcr.20170054

125. Lee A, Eifer D, Nguyen ET. A rare case of portosystemic shunt: Variation of Abernethy malformation. Journal of Cardiovascular Computed Tomography. 2018;12(2):168-169. doi:10.1016/j.jcct.2017.11.010

126. Kroencke T, Murnauer M, Jordan FA, et al. Radioembolization for Hepatocellular Carcinoma Arising in the Setting of a Congenital Extrahepatic Portosystemic Shunt (Abernethy Malformation). CardioVascular and Interventional Radiology. 2018;41(8):1285-1290. doi:10.1007/s00270-018-1965-5

127. Kang Z, Min X, Wang L. Abernethy Malformation Type II and Concurrent Nodular Hyperplasia in a Rare Female Case. Case reports in radiology. 2018;2018:6501490. doi:10.1155/2018/6501490

128. David A, Meyer J, Archambeaud I, Frampas É, Perret C, Douane F. Endovascular Closure of a Large Congenital Extrahepatic Portosystemic Shunt (Abernethy Malformation) Using Vena Cava Filter and AMPLATZER Vascular Plugs. Journal of Vascular and Interventional Radiology. 2018;29(11):1631-1633. doi:10.1016/j.jvir.2018.05.021

129. Christou N, Dib N, Chuffart E, et al. Stepwise management of hepatocellular carcinoma associated with Abernethy syndrome. Clinical Case Reports. 2018;6(5):930-934. doi:10.1002/ccr3.1384

130. Wang XY, Chen ZY, Yang YM. An Unusual Cause of Hematochezia Abernethy Malformation Type II. Gastroenterology. 2017;153(3):E3-E4. doi:10.1053/j.gastro.2017.01.039

131. Zhang XL, Duan XM, Wang FY, et al. An infant with abernethy malformation associated with heterotaxy and pulmonary hypertension. Chinese Medical Journal. 2017;130(18):2257-2258. doi:10.4103/0366-6999.213978

132. Yilmaz C, Onen Z, Farajov R, et al. Live donor liver transplantation for a child presented with severe hepatopulmonary syndrome and nodular liver lesions due to Abernethy malformation. Pediatric Transplantation. 2017;21(2). doi:10.1111/petr.12874

133. Tan Y, Guo W. Congenital extrahepatic portosystemic shunt: Report of two cases with literature review. Journal of Interventional Radiology (China). 2017;26(10):958-960. doi:10.3969/j.issn.1008-794X.2017.10.023

134. Shah A, Aziz A, Awwad A, Ramjas G, Higashi Y. Incidental radiological diagnosis of asymptomatic Abernethy malformations-two case reports. BJR case reports. 2017;3(1):20150496. doi:10.1259/bjrcr.20150496

135. Özden İ, Yavru A, Güllüoğlu M, et al. Transplantation for large liver tumors in the setting of abernethy malformation. Experimental and Clinical Transplantation. 2017;15:82-85. doi:10.6002/ect.TOND16.L23

136. Mi XX, Li XG, Wang ZR, Lin L, Xu CH, Shi JP. Abernethy malformation associated with Caroli’s syndrome in a patient with a PKHD1 mutation: a case report. Diagnostic pathology. 2017;12(1):61. doi:10.1186/s13000-017-0647-y

137. Li K, Zhai S. Abernethy malformation with abdominal pain as initial symptom: Case report. Chinese Journal of Interventional Imaging and Therapy. 2017;14(8):518. doi:10.13929/j.1672-8475.201611020

138. Li H, Ma Z, Xie Y, Tian F. Recurrent Hyperammonemia After Abernethy Malformation Type 2 Closure: a Case Report. Annals of Hepatology. 2017;16(3):460-464. doi:10.5604/01.3001.0009.8603

139. Lee SH, Yun SJ. A Type II Congenital Extrahepatic Portosystemic Shunt: A Rare Cause of Abdominal Pain Diagnosed by Point-of-Care Ultrasound. Journal of Emergency Medicine. 2017;52(6):880-882. doi:10.1016/j.jemermed.2016.12.038

140. Landau E, Shilo D, Rolnick B, Neuman J. Rare association of progressive congenital extrahepatic portosystemic shunt with development of multifocal hepatocellular carcinoma. BJR case reports. 2017;3(1):20150077. doi:10.1259/bjrcr.20150077

141. Goto K, Tsuji K, Honda Y, et al. Familial occurrence of a congenital portosystemic shunt of the portal vein. Hiroshima Journal of Medical Sciences. 2017;66(3):85-90.

142. Correa C, Luengas JP, Howard SC, Veintemilla G. Hepatoblastoma and abernethy malformation type I: Case report. Journal of Pediatric Hematology/Oncology. 2017;39(2):e79-e81. doi:10.1097/MPH.0000000000000650

143. Chira RI, Calauz A, Manole S, Valean S, Mircea PA. Unusual discovery after an examination for abdominal pain: Abernethy 1b malformation and liver adenomatosis. A case report. Journal of Gastrointestinal and Liver Diseases. 2017;26(1):85-88. doi:10.15403/jgld.2014.1121.261.abe

144. Chick JFB, Reddy SN, Yu AC, et al. Three-Dimensional Printing Facilitates Successful Endovascular Closure of a Type II Abernethy Malformation Using an Amplatzer Atrial Septal Occluder Device. Annals of Vascular Surgery. 2017;43:311.e15-311.e23. doi:10.1016/j.avsg.2017.02.012

145. Benedict M, Rodriguez-Davalos M, Emre S, Walther Z, Morotti R. Congenital extrahepatic portosystemic shunt (abernethy malformation type Ib) With associated hepatocellular carcinoma: Case report and literature review. Pediatric and Developmental Pathology. 2017;20(4):354-362. doi:10.1177/1093526616686458

146. Alharbi A, Abdulrahman S, AlOtaibi M, Alomrani A, Arabi M. Congenital Extrahepatic Portosystemic Shunt Embolization with the Use of a Duct Occluder in a Neonate with Liver Dysfunction and Hyperammonemia. Journal of Vascular and Interventional Radiology. 2017;28(9):1291-1293. doi:10.1016/j.jvir.2017.05.016

147. Sorkin T, Strautnieks S, Foskett P, et al. Multiple β-catenin mutations in hepatocellular lesions arising in Abernethy malformation. Human Pathology. 2016;53:153-158. doi:10.1016/j.humpath.2016.02.025

148. Sadat KMA. Role of ultrasonography in early diagnosis of congenital extrahepatic portosystemic shunt. BJR case reports. 2016;2(2). doi:10.1259/bjrcr.20150266

149. Merola E, Cao M, La Starza S, et al. Portosystemic encephalopathy in an 86-year-old patient: A clinical challenge. Acta Gastro-Enterologica Belgica. 2016;79(1):58-59.

150. Lautz TB, Shah SA, Superina RA. Hepatoblastoma in children with congenital portosystemic shunts. Journal of Pediatric Gastroenterology and Nutrition. 2016;62(4):542-545. doi:10.1097/MPG.0000000000001012

151. Lal BB, Arora A, Sood V, Rawat D, Khanna R, Alam S. Congenital Extrahepatic Portosystemic Shunt in a Child with Caroli’s Syndrome. ACG Case Reports Journal. 2016;3(1). doi:10.14309/CRJ.2016.164

152. Kopeć J, Krzanowski M, Królczyk J, Jaworek-Troć J, Krzanowska K, Sułowicz W. Abernethy malformation type i (congenital absence of the portal vein) in a patient with chronic kidney disease. Polskie Archiwum Medycyny Wewnetrznej. 2016;126(12):1012-1013. doi:10.20452/pamw.3729

153. Ito T, Fujimoto N, Taniguchi M, Ito M. Congenital extrahepatic portosystemic shunt with multiple visceral aneurysms. Internal Medicine. 2016;55(2):207. doi:10.2169/internalmedicine.55.5383

154. Happaerts S, Foucault A, Billiard JS, Nguyen B, Vandenbroucke-Menu F. Combined hepatocellular-cholangiocarcinoma in a patient with Abernethy malformation and tetralogy of Fallot: A case report. Hepatology. 2016;64(5):1800-1802. doi:10.1002/hep.28656

155. Gülşen Z, Yiğit H, Demir P. Multiple regenerative nodular hyperplasia in the left infrarenal vena cava accompanied by abernethy malformation. Surgical and Radiologic Anatomy. 2016;38(3):373-378. doi:10.1007/s00276-015-1460-5

156. Fu L, Wang Q, Wu J, et al. Congenital extrahepatic portosystemic shunt: an underdiagnosed but treatable cause of hepatopulmonary syndrome. European Journal of Pediatrics. 2016;175(2):195-201. doi:10.1007/s00431-015-2623-4

157. Laborda A, Guirola JA, Medrano J, Simón M, Ioakeim I, de-Gregorio M. TIPS treatment in a patient with severe lower gastrointestinal bleeding with a misdiagnosis of cirrhotic portal hypertension. Revista Espanola de Enfermedades Digestivas. 2015;107(12):766-767. doi:10.17235/reed.2015.3794/2015

158. Niyar R, Wells T, Haboubi H. Congenital extrahepatic portosystemic shunt. British Journal of Hospital Medicine. 2015;76(7):424-425. doi:10.12968/hmed.2015.76.7.424

159. Wang ZP, Luo SS, Yu N. Pulmonary hypertension induced by Abernethy malformation: Case report. Chinese Journal of Medical Imaging Technology. 2015;31(6):925. doi:10.13929/j.1003-3289.2015.06.033

160. Timpanaro T, Passanisi S, Sauna A, et al. Congenital portosystemic shunt: our experience. Case reports in pediatrics. 2015;2015:691618. doi:10.1155/2015/691618

161. Soni N, Ambesh P, Sunil K, Jena M. Abernethy malformation with Inferior Vena Cava stenosis: A cryptic cause of cyanosis. Indian Heart Journal. 2015;67(6):543-545. doi:10.1016/j.ihj.2015.06.040

162. Sinakos E, Hytiroglou P, Chourmouzi D, Akriviadis E. Focal nodular hyperplasia in an individual with Abernethy malformation type 1b. Digestive and Liver Disease. 2015;47(12):1089. doi:10.1016/j.dld.2015.07.046

163. Sharma R, Suddle A, Quaglia A, et al. Congenital extrahepatic portosystemic shunt complicated by the development of hepatocellular carcinoma. Hepatobiliary and Pancreatic Diseases International. 2015;14(5):552-557. doi:10.1016/S1499-3872(15)60418-0

164. Senniappan S, Pitt K, Shah P, et al. Postprandial hyperinsulinaemic hypoglycaemia secondary to a congenital portosystemic shunt. Hormone Research in Paediatrics. 2015;83(3):217-220. doi:10.1159/000369014

165. Saulters K, Hittle K. Hepatopulmonary syndrome in the presence of abernethy malformation: a pediatric case report. Journal of pediatric health care : official publication of National Association of Pediatric Nurse Associates & Practitioners. 2015;29(1):104-107. doi:10.1016/j.pedhc.2014.05.005

166. Sahu MK, Bisoi AK, Chander NC, Agarwala S, Chauhan S. Abernethy syndrome, a rare cause of hypoxemia: A case report. Annals of Pediatric Cardiology. 2015;8(1):64-66. doi:10.4103/0974-2069.149526

167. Loomba RS, Frommelt M, Moe D, Shillingford AJ. Agenesis of the venous duct: Two cases of extrahepatic drainage of the umbilical vein and extrahepatic portosystemic shunt with a review of the literature. Cardiology in the Young. 2015;25(2):208-217. doi:10.1017/S1047951114000729

168. Kraus C, Sheynzon V, Hanna R, Weintraub J. Single Stage Endovascular Treatment of a Type 2 Abernethy Malformation: Successful Nonsurgical Outcome in a Case Report. Case reports in radiology. 2015;2015:491867. doi:10.1155/2015/491867

169. Jiang C, Ye W, Liu C, Wu W, Li Y. Surgical Ligation of Portosystemic Shunt to Resolve Severe Hematuria and Hemafecia Caused by Type II Abernethy Malformation. Annals of Vascular Surgery. 2015;29(5):1020.e11-1020.e16. doi:10.1016/j.avsg.2015.01.023

170. Hao Y, Hong X, Zhao X. Congenital absence of the portal vein associated with focal nodular hyperplasia of the liver and congenital heart disease (Abernethy malformation): A case report and literature review. Oncology Letters. 2015;9(2):695-700. doi:10.3892/ol.2014.2767

171. Elnekave E, Belenky E, Van der Veer L. Noncirrhotic Extrahepatic Portosystemic Shunt Causing Adult-Onset Encephalopathy Treated with Endovascular Closure. Case reports in radiology. 2015;2015:852853. doi:10.1155/2015/852853

172. Brasoveanu V, Ionescu MI, Grigorie R, et al. Living donor liver transplantation for unresectable liver adenomatosis associated with congenital absence of portal vein: A case report and literature review. American Journal of Case Reports. 2015;16:637-644. doi:10.12659/AJCR.895235

173. Bas S, Guran T, Atay Z, et al. Premature pubarche, hyperinsulinemia and hypothyroxinemia: novel manifestations of congenital portosystemic shunts (Abernethy malformation) in children. Hormone Research in Paediatrics. 2015;83(4):282-287. doi:10.1159/000369395

174. Awais M, Rehman A, Baloch NUA. Congenital Absence of Portal Vein with Cecal Varices. Journal of Clinical Gastroenterology. 2015;49(7):629. doi:10.1097/MCG.0000000000000341

175. Jagadisan B, Krishnamurthy S, Raghavan R, Barathi SD. Chronic hypoxemia in a child: thinking outside the box. Indian pediatrics. 2014;51(10):829-830. doi:10.1007/s13312-014-0510-0

176. Sood V, Khanna R, Alam S, Rawat D, Bhatnagar S, Rastogi A. Ductal paucity and Warkany syndrome in a patient with congenital extrahepatic portocaval shunt. World journal of hepatology. 2014;6(5):358-362. doi:10.4254/wjh.v6.i5.358

177. Venkateshwaran S, Krishnamoorthy KM, Sivasankaran S. Percutaneous device closure of Abernethy malformation--a treatable cause of hepatopulmonary syndrome. Catheterization and cardiovascular interventions : official journal of the Society for Cardiac Angiography & Interventions. 2014;83(6):968-970. doi:10.1002/ccd.25275

178. Zhang K, Wang Q, Wang H, Ye H, Guo A, Duan W. Computed tomography and magnetic resonance imaging of multiple focal nodular hyperplasias of the liver with congenital absence of the portal vein in a Chinese girl: case report and review of the literature. European journal of medical research. 2014;19:63. doi:10.1186/s40001-014-0063-7

179. Yi JE, Jung HO, Youn HJ, Choi JY, Chun HJ, Lee JY. A case of pulmonary arterial hypertension associated with congenital extrahepatic portocaval shunt. Journal of Korean medical science. 2014;29(4):604-608. doi:10.3346/jkms.2014.29.4.604

180. Takazawa S, Uchida H, Kawashima H, et al. Massive hemorrhage after Kasai portoenterostomy in a patient with a congenital extrahepatic portosystemic shunt, malrotation and a double aortic arch: Report of a case. Surgery Today. 2014;44(8):1561-1564. doi:10.1007/s00595-013-0605-6

181. Lu J, Lin Z, Liu H, Liu Z. An unusual presentation of type II abernethy malformation. Annals of Vascular Surgery. 2014;28(6):1567.e1-1567.e4. doi:10.1016/j.avsg.2014.01.017

182. Kwapisz L, Wells MM, Al Judaibi B. Abernethy malformation: Congenital absence of the portal vein. Canadian Journal of Gastroenterology and Hepatology. 2014;28(11):587-588. doi:10.1155/2014/675812

183. Gordon-Burroughs S, Balogh J, Weiner MA, et al. Liver transplantation in an adult with adenomatosis and congenital absence of the portal vein: A case report. Transplantation Proceedings. 2014;46(7):2418-2421. doi:10.1016/j.transproceed.2014.04.012

184. Mouli VP, Gamnagatti S, Ahuja V. An Unusual Case of Cyanosis in a Young Boy. Gastroenterology. 2013;145(6):E5-E6. doi:10.1053/j.gastro.2013.07.004

185. Velayutham P, Dev A, Arora A. Congential Extrahepatic Portocaval Shunt: Growth in Vain. Journal of Pediatrics. 2013;162(5):1076-U246. doi:10.1016/j.jpeds.2012.11.066

186. Spruijt OA, Bogaard HJ, Vonk-Noordegraaf A. Pulmonary arterial hypertension combined with a high cardiac output state: Three remarkable cases. Pulmonary Circulation. 2013;3(2):440-443. doi:10.4103/2045-8932.113185

187. Yalin K, Hongyi Z, Chengli L, et al. Abernethy malformation with multiple aneurysms: incidentally found in an adult woman with Caroli’s disease. Annals of Hepatology. 2013;12(2):327-331. doi:10.1016/S1665-2681(19)31373-0

188. Tateishi Y, Furuya M, Kondo F, et al. Hepatocyte nuclear factor-1 α inactivated hepatocellular adenomas in patient with congenital absence of the portal vein: A case report. Pathology International. 2013;63(7):358-363. doi:10.1111/pin.12072

189. Schaeffer DF, Laiq S, Jang HJ, John R, Adeyi OA. Abernethy malformation type II with nephrotic syndrome and other multisystemic presentation: An illustrative case for understanding pathogenesis of extrahepatic complication of congenital portosystemic shunt. Human Pathology. 2013;44(3):432-437. doi:10.1016/j.humpath.2012.08.018

190. Raghuram KA, Bijulal S, Krishnamoorthy KM, Tharakan JA. Regression of pulmonary vascular disease after therapy of abernethy malformation in visceral heterotaxy. Pediatric Cardiology. 2013;34(8):1882-1885. doi:10.1007/s00246-012-0428-z

191. Ogul H, Bayraktutan U, Yalcin A, et al. Congenital absence of the portal vein in a patient with multiple vascular anomalies. Surgical and Radiologic Anatomy. 2013;35(6):529-534. doi:10.1007/s00276-012-1059-z

192. Ferreira A, Pereira P, Rolanda C. Diffuse Hepatic Metastasis - or Not? Abernethy Malformation Type 2 With Hepatic Nodular Regenerative Hyperplasia. Gastroenterology. 2012;142(5):1070-1257. doi:10.1053/j.gastro.2011.09.031

193. Witjes CDM, Ijzermans JNM, Noordegraaf AV, Tran TCK. Management strategy after diagnosis of Abernethy malformation: A case report. Journal of Medical Case Reports. 2012;6. doi:10.1186/1752-1947-6-167

194. Uchida H, Sakamoto S, Shigeta T, et al. Living donor liver transplantation with renoportal anastomosis for a patient with congenital absence of the portal vein. Case reports in surgery. 2012;2012:670289. doi:10.1155/2012/670289

195. Scheuermann U, Foltys D, Otto G. Focal nodular hyperplasia proceeds hepatocellular carcinoma in an adult with congenital absence of the portal vein. Transplant International. 2012;25(5):e67-e68. doi:10.1111/j.1432-2277.2012.01454.x

196. Pathak A, Agarwal N, Mandliya J, Gehlot P, Dhaneria M. Abernethy malformation: a case report. BMC Pediatrics. 2012;12. doi:10.1186/1471-2431-12-57

197. Passalacqua M, Lie KT, Yarmohammadi H. Congenital extrahepatic portosystemic shunt (Abernethy malformation) treated endovascularly with vascular plug shunt closure. Pediatric Surgery International. 2012;28(1):79-83. doi:10.1007/s00383-011-2944-y

198. Loomba RS, Telega GW, Gudausky TM. Type 2 Abernethy malformation presenting as a portal vein-coronary sinus fistula. Journal of Pediatric Surgery. 2012;47(5):E25-E31. doi:10.1016/j.jpedsurg.2011.12.031

199. Caputo S, Manganiello CAT. Severe portopulmonary hypertension associated with an unusual pattern of abernethy malformation: Three-dimensional computed tomography view. Echocardiography. 2012;29(10):E275-E277. doi:10.1111/j.1540-8175.2012.01816.x

200. Badea R, Serban A, Procopet B, Caraiani C, Pascu O. Hepatobiliary and Pancreatic: Abernethy malformation-congenital portocaval shunt. Journal of Gastroenterology and Hepatology (Australia). 2012;27(12):1875-1875. doi:10.1111/j.1440-1746.2012.07226.x

201. Asran MK, Loyer EM, Kaur H, Choi H. Case 177: Congenital absence of the portal vein with hepatic adenomatosis. Radiology. 2012;262(1):364-367. doi:10.1148/radiol.11102046

202. Chandrashekhara SH, Bhalla AS, Gupta AK, Vikash CS, Kabra SK. Abernethy malformation with portal vein aneurysm in a child. Journal of Indian Association of Pediatric Surgeons. 2011;16(1):21-23. doi:10.4103/0971-9261.74517

203. Tannuri U, Galvão F, Leal AJG, Gibelli NE, Tannuri AC. Congenital absence of the portal vein: A complex disease with multiple manifestations and types of treatment. European Journal of Pediatric Surgery. 2011;21(4):269-271. doi:10.1055/s-0031-1275675

204. Osorio MJ, Bonow A, Bond GJ, et al. Abernethy malformation complicated by hepatopulmonary syndrome and a liver mass successfully treated by liver transplantation. Pediatric Transplantation. 2011;15(7):E149-E151. doi:10.1111/j.1399-3046.2010.01337.x

205. Law YM, MacK CL, Sokol RJ, Rice M, Parsley L, Ivy D. Cardiopulmonary manifestations of portovenous shunts from congenital absence of the portal vein: Pulmonary hypertension and pulmonary vascular dilatation. Pediatric Transplantation. 2011;15(8):E162-E168. doi:10.1111/j.1399-3046.2010.01355.x

206. Kobayashi D, Edwards HD, Singh J, Nadkarni MD, Lantz PE, Cook AL. Portopulmonary hypertension secondary to congenital extrahepatic portosystemic shunt with heterotaxy and polysplenia: A cause of sudden death in an infant. Pediatric Pulmonology. 2011;46(10):1041-1044. doi:10.1002/ppul.21463

207. Kitzing YX, Gallagher J, Waugh R. Large focal nodular hyperplasia and extrahepatic portosystemic shunt in a male patient: Multi-modality imaging features. Journal of Medical Imaging and Radiation Oncology. 2011;55(5):502-505. doi:10.1111/j.1754-9485.2011.02303.x

208. Hori T, Yonekawa Y, Okamoto S, et al. Pediatric orthotopic living-donor liver transplantation cures pulmonary hypertension caused by Abernethy malformation type Ib. Pediatric Transplantation. 2011;15(3):e47-e52. doi:10.1111/j.1399-3046.2009.01269.x

209. Gadodia A, Sharma R, Kandpal H, Prashad R. Congenital absence of portal vein with large inferior mesenteric-caval shunt. Tropical gastroenterology : official journal of the Digestive Diseases Foundation. 2011;32(3):223-226.

210. Ding XY, Chen F, Zhao XX, Wu H, Chen SP, Qin YW. A rare cause of cyanosis: hepatopulmonary syndrome caused by congenital extrahepatic portosystemic shunt. Case reports in vascular medicine. 2011;2011:508171-508171. doi:10.1155/2011/508171

211. Chandler TM, Heran MK, Chang SD, Parvez A, Harris AC. Multiple focal nodular hyperplasia lesions of the liver associated with congenital absence of the portal vein. Magnetic Resonance Imaging. 2011;29(6):881-886. doi:10.1016/j.mri.2011.03.001

212. Barchetti F, Pellegrino L, Al-Ansari N, De Marco V, Scarpato P, Ialongo P. Congenital absence of the portal vein in a middle-aged man. Surgical and Radiologic Anatomy. 2011;33(4):369-372. doi:10.1007/s00276-010-0711-8

213. Banz V, Olliff S, Taniere P, Mayer D, Isaac J. Liver tumours in patients with Abernethy malformation. ANZ Journal of Surgery. 2011;81(9):640-641. doi:10.1111/j.1445-2197.2011.05842.x

214. Mistinova J, Valacsai F, Varga I. Congenital absence of the portal vein - Case report and a review of literature. Clinical Anatomy. 2010;23(7):750-758. doi:10.1002/ca.21007

215. Matsuura T, Soejima Y, Taguchi T. Auxiliary partial orthotopic living donor liver transplantation with a small-for-size graft for congenital absence of the portal vein. Liver Transplantation. 2010;16(12):1437-1439. doi:10.1002/lt.22179

216. Kuo MD, Miller FJ, Lavine JE, Peterson M, Finch M. Exploiting Phenotypic Plasticity for the Treatment of Hepatopulmonary Shunting in Abernethy Malformation. Journal of Vascular and Interventional Radiology. 2010;21(6):917-922. doi:10.1016/j.jvir.2010.01.038

217. Iida T, Ogura Y, Doi H, et al. Successful treatment of pulmonary hypertension secondary to congenital extrahepatic portocaval shunts (Abernethy type 2) by living donor liver transplantation after surgical shunt ligation. Transplant International. 2010;23(1):105-109. doi:10.1111/j.1432-2277.2009.00964.x

218. Hua J, Zhang Y, Mou W. Congenital absence of portal vein: One case report. Chinese Journal of Radiology. 2010;44(11):1218-1219. doi:10.3760/cma.j.issn.1005-1201.2010.11.028

219. Caruso S, Riva S, Spada M, Luca A, Gridelli B. Congenital Extrahepatic Portosystemic shunts (CEPS) Type Ib: MDCT finding. Digestive and Liver Disease. 2010;42(8):593. doi:10.1016/j.dld.2009.02.053

220. Afzal S, Nair A, Grainger J, Latif S, Rehman AU. Spontaneous thrombosis of congenital extrahepatic portosystemic shunt (abernethy malformation) simulating inguinal hernia incarceration. Vascular and Endovascular Surgery. 2010;44(6):508-510. doi:10.1177/1538574410373666

221. O’Leary JG, Rees CR, Klintmalm GB, Davis GL. Inferior Vena Cava Stent Resolves Hepatopulmonary Syndrome in an Adult with a Spontaneous Inferior Vena Cava-Portal Vein Shunt. Liver Transplantation. 2009;15(12):1897-1900. doi:10.1002/lt.21884

222. Tsutsui M, Sugahara S, Motosuneya T, et al. Anesthetic management of a child with Costello syndrome complicated by congenital absence of the portal vein--a case report. Paediatric anaesthesia. 2009;19(7):714-715. doi:10.1111/j.1460-9592.2009.03050.x

223. Sista AK, Filly RA. Type 1 congenital extrahepatic portosystemic shunt. Journal of Ultrasound in Medicine. 2009;28(5):703-705. doi:10.7863/jum.2009.28.5.703

224. Singhal A, Srivastava A, Goyal N, et al. Successful living donor liver transplant in a child with Abernethy malformation with biliary atresia, ventricular septal defect and intrapulmonary shunting. Pediatric Transplantation. 2009;13(8):1041-1047. doi:10.1111/j.1399-3046.2009.01092.x

225. Sanada Y, Mizuta K, Kawano Y, et al. Living Donor Liver Transplantation for Congenital Absence of the Portal Vein. Transplantation Proceedings. 2009;41(10):4214-4219. doi:10.1016/j.transproceed.2009.08.080

226. Kasahara M, Nakagawa A, Sakamoto S, et al. Living donor liver transplantation for congenital absence of the portal vein with situs inversus. Liver Transplantation. 2009;15(11):1641-1643. doi:10.1002/lt.21839

227. Hino T, Hayashida A, Okahashi N, et al. Portopulmonary hypertension associated with congenital absence of the portal vein treated with bosentan. Internal Medicine. 2009;48(8):597-600. doi:10.2169/internalmedicine.48.1715

228. Bazzocchi G, Pastorelli D, Laviani F, Simonetti G. New type of asymptomatic congenital portosystemic shunt. Clinical Journal of Gastroenterology. 2009;2(1):43-46. doi:10.1007/s12328-008-0037-9

229. Zandieh S, Vakli-Adli A, Hochreiter J, Grill F, Klaushofer K, Al Kaissi A. Ball and socket ankle joint in connection with bilateral tarsal synostosis in a boy with congenital absence of the portal vain: a novel malformation complex. Cases journal. 2008;1(1):76. doi:10.1186/1757-1626-1-76

230. Sze DY, Berquist WE. SIR 2008 Annual Meeting Film Panel Case: Abernethy Malformation. Journal of Vascular and Interventional Radiology. 2008;19(9):1274-1277. doi:10.1016/j.jvir.2008.04.017

231. Singhal M, Lal A, Thapa BR, Prakash M, Shanbhogue KP, Khandelwal N. Congenital atresia of portal vein with portocaval shunt associated with cardiac defects, skeletal deformities, and skin lesions in a boy. Journal of Pediatric Surgery. 2008;43(8):e25-e28. doi:10.1016/j.jpedsurg.2008.04.016

232. Morikawa N, Honna T, Kuroda T, et al. Resolution of hepatopulmonary syndrome after ligation of a portosystemic shunt in a pediatric patient with an Abernethy malformation. Journal of Pediatric Surgery. 2008;43(2):e35-e38. doi:10.1016/j.jpedsurg.2007.11.001

233. Luo YY, Ma M, Wang PX. Abernethy malformation in a case. Zhonghua er ke za zhi Chinese journal of pediatrics. 2008;46(12):937-938.

234. Kum Rae Kim, Jae Woon Kim. Congenital Absence of the Portal Vein in an Adult Man Presenting with Portosystemic Encephalopathy: A Case Report. Journal of the Korean Society of Radiology (JKSR). 2008;58(2):155-158.

235. Hoover W, Ackerman V, Schamberger M, Kumar M, Marshalleck F, Hoyer M. The congenital porto-caval fistula: A unique presentation and novel intervention. Pediatric Pulmonology. 2008;43(2):196-199. doi:10.1002/ppul.20727

236. Bogaard HJ, Grotjohan HP, Tjwa E, et al. A 31-year-old man with Hemoptysis at high altitude and abnormal hepatic biochemistry tests. Chest. 2007;132(3):1088-1092. doi:10.1378/chest.07-0086

237. Ratnasamy C, Kurbegov A, Swaminathan S. Cardiac anomalies in the setting of the Abernethy malformation of the portal vein. Cardiology in the Young. 2007;17(2):212-214. doi:10.1017/S1047951106001375

238. Morotti RA, Killackey M, Shneider BL, Repucci A, Emre S, Thung SN. Hepatocellular carcinoma and congenital absence of the portal vein in a child receiving growth hormone therapy for Turner syndrome. Seminars in Liver Disease. 2007;27(4):427-431. doi:10.1055/s-2007-991518

239. Kawano S, Hasegawa S, Urushihara N, et al. Hepatoblastoma with congenital absence of the portal vein - A case report. European Journal of Pediatric Surgery. 2007;17(4):292-294. doi:10.1055/s-2007-965448

240. Jun S, Lee W, Cheon JE, Kim WS, Kim IO, Yeon KM. Congenital Absence of the Portal Vein Presenting as Pulmonary Hypertension. Journal of the Korean Society of Radiology (JKSR). 2007;57(5):423-428.

241. Goo HW. Extrahepatic portosystemic shunt in congenital absence of the portal vein depicted by time-resolved contrast-enhanced MR angiography. Pediatric Radiology. 2007;37(7):706-709. doi:10.1007/s00247-007-0475-4

242. Gocmen R, Akhan O, Talim B. Congenital absence of the portal vein associated with congenital hepatic fibrosis. Pediatric Radiology. 2007;37(9):920-924. doi:10.1007/s00247-007-0533-y

243. Emre S, Amon R, Cohen E, Morotti RA, Vaysman D, Shneider BL. Resolution of hepatopulmonary syndrome after auxiliary partial orthotopic liver transplantation in abernethy malformation. A case report. Liver Transplantation. 2007;13(12):1662-1668. doi:10.1002/lt.21349

244. Alewine TC, Carter WR, Frew MJ. Congenital absence of the portal vein in a patient with urolithiasis. AJR American journal of roentgenology. 2007;189(3):W150-152. doi:10.2214/AJR.05.0843

245. Turkbey B, Karcaaltincaba M, Demir H, Akcoren Z, Yuce A, Haliloglu M. Multiple hyperplastic nodules in the liver with congenital absence of portal vein: MRI findings. Pediatric Radiology. 2006;36(5):445-448. doi:10.1007/s00247-005-0103-0

246. Tercier S, Delarue A, Rouault F, Roman C, Bréaud J, Petit P. Congenital portocaval fistula associated with hepatopulmonary syndrome: Ligation vs liver transplantation. Journal of Pediatric Surgery. 2006;41(2):E1-E3. doi:10.1016/j.jpedsurg.2005.10.071

247. Sumida W, Kaneko K, Ogura Y, et al. Living donor liver transplantation for congenital absence of the portal vein in a child with cardiac failure. Journal of Pediatric Surgery. 2006;41(11):e9-e12. doi:10.1016/j.jpedsurg.2006.07.014

248. Soejima Y, Taguchi T, Ogita K, et al. Auxiliary partial orthotopic living donor liver transplantation for a child with congenital absence of the portal vein. Liver Transplantation. 2006;12(5):845-849. doi:10.1002/lt.20692

249. Noe JA, Pittman HC, Burton EM. Congenital absence of the portal vein in a child with Turner syndrome. Pediatric Radiology. 2006;36(6):566-568. doi:10.1007/s00247-006-0169-3

250. Koizumi J, Yamashita T, Dowaki S, et al. Hepatobiliary and pancreatic: Hepatic adenoma, focal nodular hyperplasia and congenital absence of the portal vein. Journal of Gastroenterology and Hepatology (Australia). 2006;21(3):619. doi:10.1111/j.1440-1746.2006.04344.x

251. Collard B, Maleux G, Heye S, et al. Value of carbon dioxide wedged venography and transvenous liver biopsy in the definitive diagnosis of Abernethy malformation. Abdominal Imaging. 2006;31(3):315-319. doi:10.1007/s00261-005-0151-9

252. Tsuji K, Naoki K, Tachiyama Y, et al. A case of congenital absence of the portal vein. Hepatology Research. 2005;31(1):43-47. doi:10.1016/j.hepres.2004.11.006

253. Takeichi T, Okajima H, Suda H, et al. Living domino liver transplantation in an adult with congenital absence of portal vein. Liver Transplantation. 2005;11(10):1285-1288. doi:10.1002/lt.20561

254. Ohnishi Y, Ueda M, Doi H, et al. Successful liver transplantation for congenital absence of the portal vein complicated by intrapulmonary shunt and brain abscess. Journal of Pediatric Surgery. 2005;40(5):E1-E3. doi:10.1016/j.jpedsurg.2005.02.011

255. Kornprat P, Langner C, Fritz K, Mischinger HJ. Congenital absence of the portal vein in an adult woman: A case report. Wiener Klinische Wochenschrift. 2005;117(1-2):58-62. doi:10.1007/s00508-004-0281-z

256. Dahlan M, Situmorang I. Congenital absence of the portal vein with splenomegaly in a young woman (case report). Medical Journal of Indonesia. 2005;14(2):122-127. doi:10.13181/mji.v14i2.177

257. Cheung KM, Lee CY, Wong CT, Chan AKH. Congenital absence of portal vein presenting as hepatopulmonary syndrome. Journal of Paediatrics and Child Health. 2005;41(1-2):72-75. doi:10.1111/j.1440-1754.2005.00542.x

258. Kinane TB, Westra SJ. A four-year-old boy with hypoxemia - Hepatopulmonary syndrome due to the Abernethy malformation, type 1. New England Journal of Medicine. 2004;351(16):1667-1675. doi:10.1056/NEJMcpc049023

259. Wojcicki M, Haagsma EB, Gouw ASH, Slooff MJH, Porte RJ. Orthotopic liver transplantation for portosystemic encephalopathy in an adult with congenital absence of the portal vein. Liver Transplantation. 2004;10(9):1203-1207. doi:10.1002/lt.20170

260. Takagaki K, Kodaira M, Kuriyama S, et al. Congenital absence of the portal vein complicating hepatic tumors. Internal Medicine. 2004;43(3):194-198. doi:10.2169/internalmedicine.43.194

261. Manning N, Impey L, Lindsell D, Lakhoo K. Prenatally diagnosed portocaval shunt and postnatal outcome: A case report. Prenatal Diagnosis. 2004;24(7):537-540. doi:10.1002/pd.947

262. De Gaetano AM, Gui B, Macis G, Manfredi R, Di Stasi C. Congenital absence of the portal vein associated with focal nodular hyperplasia in the liver in an adult woman: Imaging and review of the literature. Abdominal Imaging. 2004;29(4):455-459. doi:10.1007/s00261-003-0131-x

263. Charre L, Roggen F, Lemaire J, et al. Hematochezia and congenital extrahepatic portocaval shunt with absent portal vein: Successful treatment by liver transplantation [1]. Transplantation. 2004;78(9):1404-1406. doi:10.1097/01.TP.0000137931.51504.F7

264. Tanaka Y, Takayanagi M, Shiratori Y, et al. Congenital absence of portal vein with multiple hyperplastic nodular lesions in the liver. Journal of Gastroenterology. 2003;38(3):288-294. doi:10.1007/s005350300050

265. Pohl A, Jung A, Vielhaber H, et al. Congenital atresia of the portal vein and extrahepatic portocaval shunt associated with benign neonatal hemangiomatosis, congenital adrenal hyperplasia, and atrial septal defect. Journal of Pediatric Surgery. 2003;38(4):633-634. doi:10.1053/jpsu.2003.50140

266. Pichon N, Maisonnette F, Pichon-Lefièvre F, Valleix D, Pillegand B. Hepatocarcinoma with congenital agenesis of the portal vein. Japanese Journal of Clinical Oncology. 2003;33(6):314-316. doi:10.1093/jjco/hyg050

267. Murray CP, Yoo SJ, Babyn PS. Congenital extrahepatic portosystemic shunts. Pediatric Radiology. 2003;33(9):614-620. doi:10.1007/s00247-003-1002-x

268. Kanamori Y, Hashizume K, Kitano Y, Sugiyama M, Motoi T, Tange T. Congenital extrahepatic portocaval shunt (Abernethy type 2), huge liver mass, and patent ductus arteriosus--a case report of its rare clinical presentation in a young girl. Journal of Pediatric Surgery. 2003;38(4):E15. doi:10.1053/jpsu.2003.50153

269. Alvarez AE, Ribeiro AF, Hessel G, Baracat J, Ribeiro JD. Abernethy malformation: One of the etiologies of hepatopulmonary syndrome. Pediatric Pulmonology. 2002;34(5):391-394. doi:10.1002/ppul.10182

270. Venkat-Raman N, Murphy KW, Ghaus K, Teoh TG, Higham JM, Carvalho JS. Congenital absence of portal vein in the fetus: A case report. Ultrasound in Obstetrics and Gynecology. 2001;17(1):71-75. doi:10.1046/j.1469-0705.2001.00312.x

271. Shinkai M, Ohhama Y, Nishi T, et al. Congenital absence of the portal vein and role of liver transplantation in children. Journal of Pediatric Surgery. 2001;36(7):1026-1031. doi:10.1053/jpsu.2001.24731

272. Lundstedt C, Lindell G, Tranberg KG, Svartholm E. Congenital absence of the intrahepatic portion of the portal vein in an adult male resected for hepatocellular carcinoma. European Radiology. 2001;11(11):2228-2231. doi:10.1007/s003300100833

273. Kinjo T, Aoki H, Sunagawa H, Kinjo S, Muto Y. Congenital absence of the portal vein associated with focal nodular hyperplasia of the liver and congenital choledochal cyst: A case report. Journal of Pediatric Surgery. 2001;36(4):622-625. doi:10.1053/jpsu.2001.22303

274. Yonemitsu H, Mori H, Kimura T, et al. Congenital extrahepatic portocaval shunt associated with hepatic hyperplastic nodules in a patient with Dubin-Johnson syndrome. Abdominal Imaging. 2000;25(6):572-575. doi:10.1007/s002610000044

275. Shinkai M, Ohhama Y, Nishi T, et al. Living related partial liver transplantation for hyperammonemia due to congenital absence of the portal vein. Transplantation Proceedings. 2000;32(7):2184. doi:10.1016/S0041-1345(00)01626-2

276. Ishii Y, Inagaki Y, Hirai K, Aoki T. Hepatic encephalopathy caused by congenital extrahepatic portosystemic venous shunt. Journal of hepato-biliary-pancreatic surgery. 2000;7(5):524-528. doi:10.1007/s005340070026

277. Grazioli L, Alberti D, Olivetti L, et al. Congenital absence of portal vein with nodular regenerative hyperplasia of the liver. European Radiology. 2000;10(5):820-825. doi:10.1007/s003300051012

278. Wakamoto H, Manabe K, Kobayashi H, Hayashi M. Subclinical portal-systemic encephalopathy in a child with congenital absence of the portal vein. Brain and Development. 1999;21(6):425-428. doi:10.1016/S0387-7604(99)00049-2

279. Massin M, Verloes A, Jamblin P. Cardiac anomalies associated with congenital absence of the portal vein. Cardiology in the Young. 1999;9(5):522-525.

280. Kohda E, Saeki M, Nakano M, et al. Congenital absence of the portal vein in a boy. Pediatric Radiology. 1999;29(4):235-237. doi:10.1007/s002470050580

281. Kim SZ, Marz PL, Laor T, Teitelbaum J, Jonas MM, Levy HL. Elevated galactose in newborn screening due to congenital absence of the portal vein [4]. European Journal of Pediatrics. 1998;157(7):608-609. doi:10.1007/s004310050892

282. Guariso G, Fiorio S, Altavilla G, et al. Congenital absence of the portal vein associated with focal nodular hyperplasia of the liver and cystic dysplasia of the kidney. European Journal of Pediatrics. 1998;157(4):287-290. doi:10.1007/s004310050812

283. Altavilla G, Cusatelli P. Ultrastructural analysis of the liver with portal vein agenesis: A case report. Ultrastructural Pathology. 1998;22(6):477-483. doi:10.3109/01913129809032284

284. Motoori S, Shinozaki M, Goto N, Kondo F. Case report: Congenital absence of the portal vein associated with nodular hyperplasia in the liver. Journal of Gastroenterology and Hepatology (Australia). 1997;12(9-10):639-643. doi:10.1111/j.1440-1746.1997.tb00527.x

285. Howard ER, Davenport M. Congenital extrahepatic portocaval shunts - The Abernethy malformation. Journal of Pediatric Surgery. 1997;32(3):494-497. doi:10.1016/S0022-3468(97)90614-X

286. Kiriyama M, Takashima S, Sahara H, et al. Portal-systemic encephalopathy due to a congenital extrahepatic portosystemic shunt. Journal of Gastroenterology and Hepatology. 1996;11(7):626-629. doi:10.1111/j.1440-1746.1996.tb00304.x

287. Laverdiere JT, Laor T, Benacerraf B. CONGENITAL ABSENCE OF THE PORTAL-VEIN - CASE-REPORT AND MR DEMONSTRATION. Pediatric Radiology. 1995;25(1):52-53. doi:10.1007/bf02020848

288. Morgan G, Superina R. Congenital absence of the portal vein: Two cases and a proposed classification system for portasystemic vascular anomalies. Journal of Pediatric Surgery. 1994;29(9):1239-1241. doi:10.1016/0022-3468(94)90812-5

289. Kamiya S, Taniguchi I, Yamamoto T, et al. Analysis of intestinal flora of a patient with congenital absence of the portal vein. FEMS Immunology and Medical Microbiology. 1993;7(1):73-80. doi:10.1016/0928-8244(93)90046-7

290. Matsuoka Y, Ohtomo K, Okubo T, Nishikawa J, Mine T, Ohno S. Congenital absence of the portal vein. Gastrointestinal Radiology. 1992;17(1):31-33.

291. Nakasaki H, Tanaka Y, Ohta M, et al. Congenital absence of the portal vein. Annals of Surgery. 1989;210(2):190-193. doi:10.1097/00000658-198908000-00009

292. Barton Iii JW, Keller MS. Liver transplantation for hepatoblastoma in a child with congenital absence of the portal vein. Pediatric Radiology. 1989;20(1-2):113-114. doi:10.1007/BF02010653

293. Morse SS, Taylor KJ, Strauss EB, Ramirez E, Seashore JH. Congenital absence of the portal vein in oculoauriculovertebral dysplasia (Goldenhar syndrome). Pediatric Radiology. 1986;16(5):437-439. doi:10.1007/bf02386831

294. Yancy AG, Jeffries CP, Miller RM. Congenital absence of the portal vein. Journal of the National Medical Association. 1961;53:119-121.

295. Abernethy Malformation and Gastrointestinal Bleeding: A Case Report and Literature Review - Wenrui Li, Bin Liu, Hai Feng, 2024. Accessed September 18, 2024. https://journals.sagepub.com/doi/abs/10.1177/15385744241278870
